# A Network Meta-Analysis of Long-Acting Muscarinic Antagonist (LAMA) and Long-Acting β_2_-Agonist (LABA) Combinations in COPD

**DOI:** 10.1007/s41030-017-0048-0

**Published:** 2017-08-22

**Authors:** Katya Y. J. Sion, Eline L. Huisman, Yogesh S. Punekar, Ian Naya, Afisi S. Ismaila

**Affiliations:** 1 Real World Strategy and Analytics, Mapi Group, Houten, The Netherlands; 2 ViiV Healthcare, GlaxoSmithKline, Brentford, Hounslow, Middlesex UK; 3 Respiratory Medicine Development Centre, GlaxoSmithKline, Brentford, Hounslow, Middlesex UK; 4 Value Evidence and Outcomes, GlaxoSmithKline, 5 Moore Drive, PO Box 13398, Research Triangle Park, NC 27709-3398 USA; 5 Department of Clinical Epidemiology and Biostatistics, McMaster University, Hamilton, ON Canada; 6 Present Address: Care and Public Health Research Institute, Maastricht University, Maastricht, The Netherlands; 7 Present Address: Novo Nordisk, Alphen aan den Rijn, The Netherlands

**Keywords:** Bayesian analysis, Combination bronchodilator, Comparative efficacy, COPD, Indirect treatment comparison, LABA, LAMA, Meta-analysis

## Abstract

**Introduction:**

Comparative data on the efficacies of long-acting muscarinic antagonist (LAMA) and long-acting β_2_-agonist (LABA) combinations for the treatment of moderate-to-very-severe chronic obstructive pulmonary disease (COPD) are limited. The aim of this Bayesian network meta-analysis (NMA) is to assess the relative efficacies of available open combinations (delivered via separate inhalers) and fixed-dose combinations (FDCs, delivered via a single inhaler).

**Methods:**

We conducted a systematic literature review with the aim of identifying randomized controlled trials (RCTs) of ≥8-week duration in adults aged ≥40 years with COPD that compared LAMA + LABA combinations with each other, with tiotropium (TIO), or with placebo. Data on changes from baseline in trough forced expiratory volume in 1 s (FEV_1_) and on St George’s Respiratory Questionnaire (SGRQ) total score, the Transition Dyspnea Index (TDI) focal score, and rescue medication use at 12 and 24 weeks were extracted from these RCTs and analyzed using a NMA in a Bayesian framework.

**Results:**

Data from 44 RCTs were included in the NMA. All FDCs showed improvements relative to placebo in terms of trough FEV_1_, SGRQ total score, and TDI focal score above clinically relevant thresholds, with the exception of TIO/olodaterol and aclidinium/formoterol, both of which failed to show clinically relevant improvements in SGRQ score at 24 weeks. All FDCs demonstrated reduced rescue medication use versus placebo. Open combinations demonstrated improved efficacy in all outcomes versus placebo, but these improvements did not consistently exceed clinically relevant thresholds for SGRQ and TDI scores. All once-daily FDCs showed improved efficacy versus TIO, but improvements were less consistently observed versus TIO with open dual combinations and combinations containing formoterol or salmeterol administered twice daily. Relative probabilities of improvement between FDCs highlighted potential between-class differences for trough FEV_1_ but suggested little potential for differences in patient-reported outcomes.

**Conclusion:**

LAMA + LABA combinations generally showed improved outcomes versus placebo and TIO. FDCs appeared to perform better than open dual combinations. A potential effectiveness gradient was observed between FDCs for objectively assessed functional outcomes, although further prospective trials are required to confirm these findings.

**Funding:**

GSK.

**Electronic supplementary material:**

The online version of this article (doi:10.1007/s41030-017-0048-0) contains supplementary material, which is available to authorized users.

## Introduction

Bronchodilators, particularly long-acting muscarinic antagonists (LAMAs) and long-acting β_2_-agonists (LABAs), have become the mainstay of pharmacological therapy for chronic obstructive pulmonary disease (COPD) [1–3]. Despite the well-demonstrated utility of LABAs [4–7] and LAMAs [8–11] as maintenance therapy, a high number of patients with moderate or severe COPD receiving LABA or LAMA monotherapy can fail to achieve adequate control of symptoms [12]. A combination of a LAMA and a LABA maintenance therapy is a logical therapeutic option to improve symptom control for such patients [13]. In the last decade, multiple randomized controlled trials (RCTs) have reported that improvements in lung function and patient-reported outcomes (PROs) can be achieved with LAMA + LABA combinations compared with the component monotherapies for patients with stable COPD, with no differences in safety [14–20].

LAMA + LABA combinations can be administered as open dual combinations (using separate prescribed inhalers for each bronchodilator) and fixed-dose combinations (FDCs; use of a single inhaler delivering a fixed dose of each bronchodilator). Several options are available within each class; in the last few years, several FDC LAMA/LABA therapies have been approved as once-daily (OD) or twice-daily (BID) maintenance therapies for COPD, including glycopyrronium/indacaterol (GLY/IND) 110/50 mcg OD (outside the USA) [21] and 27.5/15.6 mcg BID (USA only) [22], umeclidinium/vilanterol (UMEC/VI) 62.5/25 mcg OD [23, 24], aclidinium/formoterol (ACL/FOR) 400/12 mcg BID [25], and tiotropium/olodaterol (TIO/OLO) 5/5 mcg OD [26, 27]. With the increasing range of LAMA + LABA therapies that have become available, it is desirable to assess their comparative efficacies and characteristics. Limited data are currently available from direct comparisons between FDC LAMA/LABA therapies or of FDC therapies with open dual LAMA + LABA combinations. There is therefore a need to synthesize the available data from RCTs to enable comparisons to be made between therapies.

The aim of this Bayesian network meta-analysis (NMA) was to compare the efficacy of available FDC and open dual LAMA + LABA bronchodilators in patients with moderate-to-very-severe COPD, based on a systematic literature review (SLR) up to October 2015. The NMA reported here contains a further 18 trials that were not previously available in an earlier NMA based on the literature up to the end of 2014 [28]. A novel aspect of this NMA compared with other NMAs in the area is the potential to examine differences in efficacy between FDCs and open dual LAMA + LABA combinations, and between once- and twice-daily therapies. An added feature was to explore subgroup analyses where possible to observe the impact of severity of lung function impairment and use of concurrent inhaled corticosteroids (ICS) across different trials on the relative efficacies of bronchodilators.

## Methods

### Data Sources

This article is based on a synthesis of previously conducted studies and does not involve any new studies of human or animal subjects performed by any of the authors.

A SLR was performed to identify RCTs comparing open or FDC LAMA + LABA therapies with each other, with TIO monotherapy, or with placebo in adult patients with COPD. A broad search strategy was utilized to include all available LAMAs, LABAs, and LAMA + LABA combinations. No time restrictions were employed. Several databases and trial registries were searched, including MEDLINE^®^, Embase^®^, and clinicaltrials.gov, in October 2015 using pre-defined search strategies specifically tailored to each platform [details on the data sources and search strategies are presented in Electronic Supplementary Material (ESM) Tables S1, S2].

### Inclusion Criteria and Study Selection Process

Abstracts were screened independently by two reviewers, with any discrepancies resolved by consensus. RCTs included in the NMA were those published in English or German, of ≥8 weeks’ duration, involving adults of ≥40 years of age with COPD as defined by the Global Initiative for Chronic Obstructive Lung Disease (GOLD) guidelines [1].

Only RCTs involving FDC LAMA/LABAs or open dual LAMA + LABA combinations, TIO 18 mcg OD (TIO 18), TIO 5 mcg OD (TIO 5), or placebo were included in the NMA. TIO monotherapy was included as a comparator as it was used as the principal comparator instead of placebo in several studies. Studies comparing TIO with placebo were also included to strengthen the network.

Included trials were required to report outcomes within a 4-week window at 12 (8–16) weeks and/or 24 (20–28) weeks. Outcomes of interest were: trough forced expiratory volume in 1 s (FEV_1_), and the subjective PROs of breathlessness assessed by the Transition Dyspnea Index (TDI) focal score, health-related quality of life (HRQoL) assessed by the St George’s Respiratory Questionnaire (SGRQ) total score, and rescue short-acting β_2_-agonist medication use (puffs/day).

### Data Extraction and Quality Assessment

Data extraction was performed by one researcher and reviewed by a second. Key data from each eligible study, including study characteristics, patient characteristics at baseline, and outcomes of interest, were extracted by recording data from the original report into a standard data extraction form. For the outcomes of interest, the mean or least squares mean difference in change from baseline (CFB) between the arms of interest, and associated 95% confidence intervals (95% CIs), standard error (SE), or standard deviation (SD), were abstracted. If not reported, the difference in CFB was calculated based on the CFB in each treatment arm, and the SE was imputed using the uncertainty of other trials in the network [29]. A checklist for assessing the risk of bias in RCTs based on guidance from the Institute for Quality and Efficiency in Health Care was employed [30].

To reduce the risk of imbalances in effect modifiers (any characteristic of patients or studies which would influence the treatment effect) across studies and obtaining biased outcomes in the NMA, the similarity of studies was assessed by evaluating the distribution of patient characteristics and study designs across the direct comparisons in the network. If any major differences were detected, subgroup analysis was used to evaluate the impact of the difference on treatment effect. Subgroup analyses were performed for four patient subgroups: ICS users and non-users, and patients with moderate or severe disease (as reported in the individual study publications). These analyses were only feasible for the outcome of trough FEV_1_ due to the limited data available for the other outcomes. It was not feasible to use meta-regression techniques to adjust for variations due to the relatively limited number of studies available in the network.

### Bayesian NMA

A NMA was performed within a Bayesian framework [31–33] to analyze the extracted data and thereby synthesize the results of the included studies and assess relative treatment effects. The details of this framework have been presented previously [28]; briefly, a generalized linear model with identity link and a normal likelihood distribution was employed, with non-informative prior distributions assumed for all outcomes.

For each outcome, fixed- and random-effects models were evaluated. The most appropriate model for each outcome was identified by calculating the Deviance Information Criterion, a measure of goodness of fit based on the posterior mean residual deviance. The parameters of the different models were estimated within a Bayesian framework using a Markov Chain Monte Carlo method implemented in the R and OpenBUGS software packages (Foundation for Statistical Computing, Vienna, Austria). The OpenBUGS codes were based on those presented by Dias et al. [34].

The Bayesian NMA provides posterior distributions of the relative treatment effects between different therapies for each outcome of interest. The posterior distributions are summarized using the median (reflecting the most likely value of the estimate) and the 2.5 and 97.5 percentiles to capture the 95% credible interval (95% CrI), with the latter representing the range of true underlying effects with a probability of 95%. For each endpoint, the probability that each treatment is better than a certain comparator is presented; the probability of the comparator being better than the initial treatment is the difference between 100 and the original probability. Studies reporting mean values without any measure of uncertainty (SE, SD, 95% CI) were included in the base case analysis with imputed SE values but excluded from scenario analyses [29]. A separate analysis without imputation was also performed. A scenario analysis was conducted to evaluate the impact of pooling TIO 18 and TIO 5 on the relative effects on trough FEV_1_.

## Results

### Study Selection

**Fig. 1:**
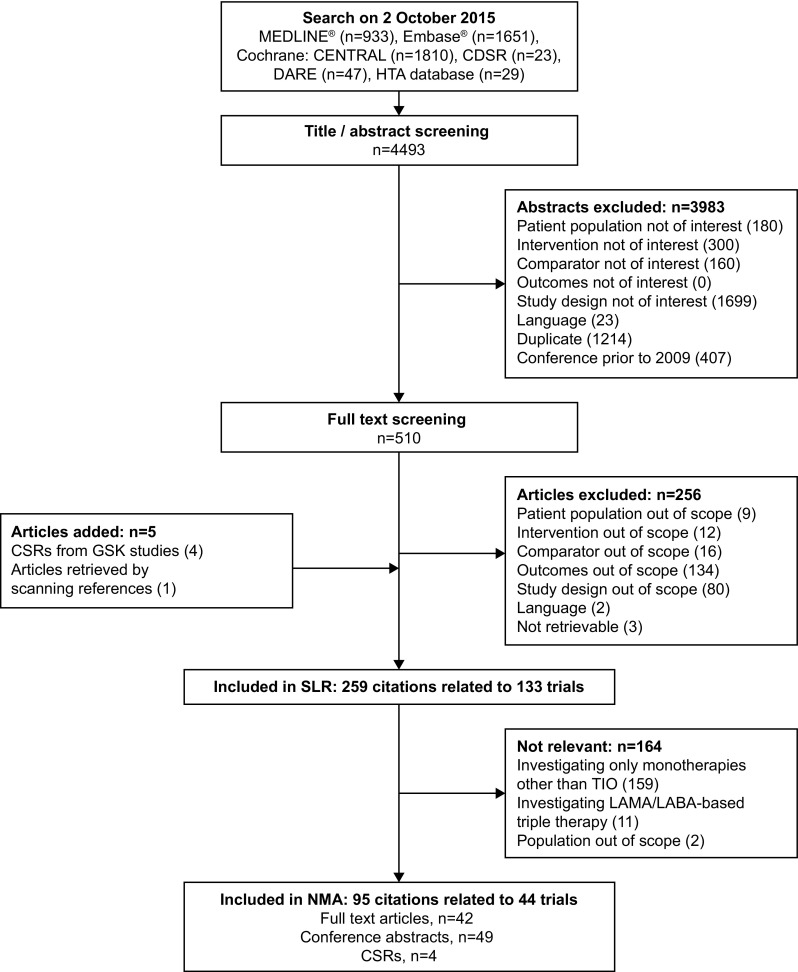
PRISMA flow diagram of study selection process. *CDSR* Cochrane Database of Systematic Reviews, *CSR* clinical study report, *DARE* Database of Abstracts of Reviews of Effects,* GSK* GlaxoSmithKline, *HTA* Health Technology Assessment, *LABA* long-acting β_2_-agonist, *LAMA* long-acting muscarinic antagonist, *NMA* Network meta-analysis, *SLR* systematic literature review, *TIO* tiotropium

Of a total of 4493 abstracts identified in the initial literature search, 510 (11.4%) were assessed to be of interest (Fig. 1). After exclusion of 256 articles, and the addition of four GlaxoSmithKline (GSK) clinical study reports and an additional article retrieved by scanning references, 259 citations were included in the SLR. Parallel online searches of trial registries identified 5838 relevant records. After exclusion of 5650 registries (2304 based on study design, 611 based on population, 997 based on intervention, 79 based on comparator, 12 based on outcomes, 13 based on language, and 1634 duplicates), 188 registries were suitable for inclusion in the SLR. After merging the searches and excluding trials involving non-relevant comparators, we identified a total of 95 citations related to 44 RCTs which were included in the NMA.

Characteristics of the included RCTs are presented in ESM Table S3, and the results of the assessment for risk of bias are summarized in ESM Table S4. Most studies were randomized, double-blind, multicenter trials. Trough FEV_1_ was the most commonly reported outcome, while rescue medication use was the least common (Table 1). The majority of trials compared TIO [either TIO 18 (18 trials) or TIO 5 (6 trials)] with placebo. Most trials had a duration of either 12–13 weeks (16 trials) or approximately 26 weeks (15 trials). Nine trials had a duration of approximately 1 year, while three trials were longer: the SPARK trial (duration 64 weeks [35]), the TIOSPIR^®^ trial (duration 2.3 years [36]), and the UPLIFT trial (duration 4 years [11]). Nearly all of the trials (40/44) allowed concomitant ICS use; concurrent LABA therapy was allowed in eight trials which compared TIO with placebo, but was not allowed in the trials involving LAMA + LABA therapy.

**Table 1:** Results of the base case Bayesian network meta-analysis: placebo-adjusted levels of efficacy of each different bronchodilator

Outcome of interest	Number of RCTs analyzed	Monotherapy (in mcg)^a^	Open dual combinations (in mcg)^a^				
		TIO 5 or 18	TIO 18 + FOR 12^b^	TIO 18 + IND 150	TIO 18 + OLO 5	TIO 18 + FOR 10^b^	TIO 18 + SAL 50^c^
Trough FEV_1_ at 12 weeks (CFB, mL)	33	114.70 (106.20, 123.40); >99%	151.60 (118.80, 183.10); >99%	188.60 (170.20, 207.30); >99%	165.70 (145.30, 186.20); >99%	NA	NA
Trough FEV_1_ at 24 weeks (CFB, mL)	24	110.90 (102.90, 118.90); >99%	113.50 (76.33, 150.40); >99%	NA	NA	NA	123.60 (39.40, 215.90); >99%
SGRQ total score at 12 weeks (CFB)	17	−2.97 (−3.41, −2.53); >99%	−3.99 (−6.94, −1.03); >99%	NA	−4.82 (−5.82, −3.82); >99%	NA	NA
SGRQ total score at 24 weeks (CFB)	20	−2.53 (−2.94, 2.12); >99%	NA	NA	NA	−3.18 (−5.46, −0.89); >99%	−4.00 (−5.42, −2.59); >99%
TDI focal score at 12 weeks	14	0.79 (0.52, 1.06); >99%	0.86 (−0.12, 1.85); 96%	NA	NA	NA	NA
TDI focal score at 24 weeks	15	0.83 (0.65, 1.01); >99%	0.93 (0.41, 1.44); >99%	NA	NA	NA	0.41 (−0.46, 1.27); 82%
Rescue medication use at 12 weeks (CFB)	11	−0.29 (−0.64, 0.06); 95%	−0.54 (−1.26, 0.17); 93%	−1.16 (−1.58, −0.74); >99%	NA	NA	NA
Rescue medication use at 24 weeks (CFB)	9	−0.65 (−1.26, −0.11); 99%	NA	NA	NA	NA	NA

Outcome of interest	Fixed-dose combinations (in mcg)^a^				
	ACL/FOR 400/12^d^	TIO/OLO 5/5	GLY/IND 27.5/15.6^d^	GLY/IND 110/50	UMEC/VI 62.5/25
Trough FEV_1_ at 12 weeks (CFB, mL)	135.90 (110.60, 160.80;) >99%	170.20 (153.50, 186.90) >99%	222.20 (193.30, 250.90); >99%	205.10 (185.70, 224.00); >99%	208.10 (187.90, 228.20); >99%
Trough FEV_1_ at 24 weeks (CFB, mL)	138.20 (117.90, 158.30); >99%	172.10 (152.70, 191.70); >99%	NA	181.80 (162.50, 200.90); >99%	195.80 (174.80, 217.30); >99%
SGRQ total score at 12 weeks (CFB)	NA	−4.90 (−6.10, −3.68); >99%	−4.97 (−6.42, −3.52); >99%	−4.89 (−5.98, −3.80); >99%	−4.70 (−5.72, −3.68); >99%
SGRQ total score at 24 weeks (CFB)	−2.80 (−4.37, −1.22); >99%	−3.76 (−4.92, −2.60); >99%	NA	−4.29 (−5.34, −3.23); >99%	−4.11 (−5.23, −2.99); >99%
TDI focal score at 12 weeks	NA	1.50 (1.01, 2.00); >99%	1.62 (1.07, 2.17); >99%	1.34 (0.67, 1.99); >99%	1.24 (0.77, 1.73); >99%
TDI focal score at 24 weeks	1.37 (0.98, 1.76); >99%	1.19 (0.87, 1.51); >99%	NA	1.31 (1.03, 1.58); >99%	1.01 (0.66, 1.36); >99%
Rescue medication use at 12 weeks (CFB)	NA	−0.97 (−1.38, −0.56); >99%	−1.19 (−1.45, −0.93); >99%	NA	−0.84 (−1.19, −0.49); >99%
Rescue medication use at 24 weeks (CFB)	−0.66 (−1.75, 0.44); 92%	−1.28 (−2.52, −0.12); 98%	NA	−1.09 (−2.07, −0.12); 98%	−1.13 (−1.86, −0.46); >99%

Results in table are reported as the mean improvement with the 95% credible interval (95% CrI) in parenthesis, followed by the percentage probability of improved outcome versus placebo

*ACL* aclidinium, *CFB* change from baseline, *CrI* credible interval, *FEV*_*1*_ forced expiratory volume in 1 s, *FOR* formoterol, *GLY* glycopyrronium, *IND* indacaterol, *NA* data not available, *NMA* network meta-analysis, *OLO* olodaterol, *RCT* randomized controlled trial, *SAL* salmeterol, *SGRQ* St George’s Respiratory Questionnaire, *TDI* Transition Dyspnea Index, *TIO* tiotropium, *UMEC* umeclidinium, *VI* vilanterol

^a^Numbers following drug are the dose. See "Introduction" for a more detailed explanation. Note: unless indicated otherwise, all doses were taken once daily.

^b^Formoterol dose taken twice daily

^c^Salmeterol dose taken twice daily

^d^Combined dose taken twice daily

### Patient Characteristics

**Fig. 2:**
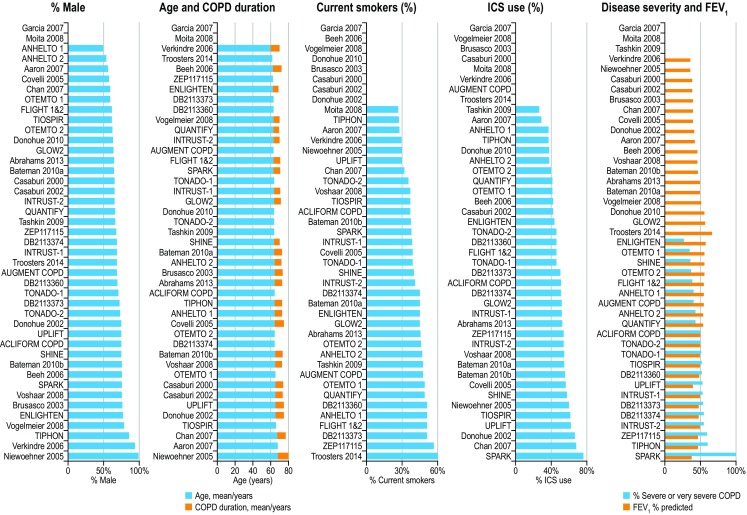
Patient characteristics in the randomized controlled trials included in the NMA. Values are weighted means of the patient populations included in the NMA. Zero values indicate that data were not reported. *COPD* chronic obstructive pulmonary disease, *FEV*_*1*_ forced expiratory volume in 1 s, *ICS* inhaled corticosteroids, *NMA* network meta-analysis

Patient characteristics for all included RCTs are presented in Fig. 2. The mean age of patients ranged between 60.5 and 67.9 years, with the percentage of males ranging between 49.5 and 98.5%. The increased variability in gender was largely attributable to three trials comparing TIO with placebo, one of which recruited patients from medical centers for veterans, who are predominantly male. Patients were current smokers (range 26.4–59.5%) or former smokers at study entry, all with a smoking history of >10 pack-years. Most trials included patients with both moderate and severe COPD (GOLD stages II and III); some also included patients with very severe disease (GOLD stage IV). The SPARK trial was a notable exception as it included only patients with severe or very severe COPD, with 76% of patients enrolled using concurrent ICS, and all patients having reported at least one moderate/severe exacerbation in the previous year (Fig. 2) [35].

### Bayesian NMA

Because many studies used TIO 18 or TIO 5 as a comparator arm instead of placebo, the base case was performed with studies comparing a LAMA + LABA intervention of interest to another LAMA + LABA intervention of interest or to TIO 18 or TIO 5, or placebo. It was assumed that the results from TIO 18 and TIO 5 were sufficiently similar to be pooled together: similar efficacy and safety have been reported for both doses in a previous NMA [37] and study [38]. A scenario analysis was conducted for trough FEV_1_ at 12 and 24 weeks to evaluate the impact of pooling the two doses.

**Fig. 3:**
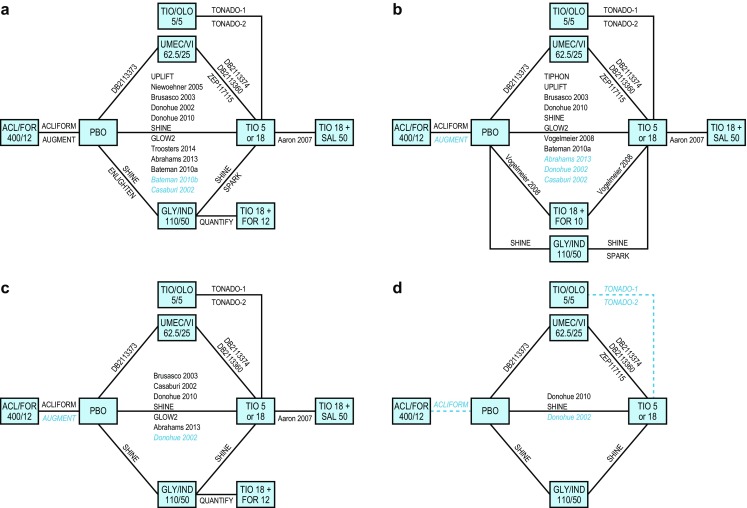
Overall network of studies in the base case NMA of LAMA + LABA combination therapies evaluated at 24 weeks for trough FEV_1_ (**a**), St George’s Respiratory Questionnaire (*SGRQ*) total score (**b**), Transition Dyspnea Index (*TDI*) focal score (**c**), and rescue medication use (**d**). Studies in *blue text* represent those that reported mean values without standard errors/standard deviations/confidence intervals. *Blue dotted lines* represent relationships for which errors were calculated by imputation. *ACL* aclidinium, *FEV*_*1*_ forced expiratory volume in 1 s, *FOR* formoterol, *GLY* glycopyrronium, *IND* indacaterol, *LABA* long-acting β_2_-agonist, *LAMA* long-acting muscarinic antagonist, *NMA* network meta-analysis, *OLO* olodaterol, *PBO* placebo, *SAL* salmeterol, *TIO *tiotropium, *UMEC* umeclidinium, *VI* vilanterol

The overall networks of studies for the base case analysis for each outcome of interest at 24 weeks are presented in Fig. 3. The individual study results for each outcome of interest are presented in ESM Table S5.

Results are presented for each of the four outcomes of interest (trough FEV_1_, SGRQ total score, TDI focal score and rescue medication use) for each time point (12 and 24 weeks). For each set of results, results of the base case NMA (with imputation) are presented first, comparing the active therapies with placebo, then the dual therapies with TIO, and finally the dual therapies with each other. Results obtained without imputation of SEs are then briefly summarized, followed by results of the scenario analysis, where applicable. Finally, results of the subgroup analyses are presented.

### NMA Results: Trough FEV_1_

#### 12 Weeks

The base case analysis results at 12 weeks showed that all active therapies achieved improvements in trough FEV_1_ versus placebo, with probabilities improved outcomes of >99% (Table 1). All improvements were greater than the minimal clinically important difference (MCID) of 100 mL [39]. In comparison with TIO, all combination therapies were superior, with probabilities of improved outcomes of ≥94% (ESM Table S6).

Comparing the combination therapies, the open combination TIO 18 OD + IND 150 OD (TIO + IND) and the FDCs UMEC/VI and GLY/IND (both doses) all had high probabilities (≥94%) of improved outcomes versus the other therapies [TIO 18 OD + FOR 12 BID, ACL/FOR, TIO 18 OD + OLO 5 OD (TIO + OLO) and TIO/OLO]. Between these three therapies, the FDCs appeared to be superior to the open combination, with probabilities of improved outcomes of ≥91%. The probability of improved outcomes using GLY/IND versus UMEC/VI was 78% for the comparison with GLY/IND 27.5/15.6, but only 42% for the comparison with GLY/IND 110/50 (ESM Table S6).

Results using data without imputed SEs were generally similar to the base case results, with only small differences (<5 mL) in changes in FEV_1_ for most comparisons except those with TIO/OLO for which differences were larger (<15 mL; data not shown). Comparisons with ACL/FOR were not included as these data were obtained by imputation.

In the scenario analysis, separating TIO 18 and TIO 5 had little impact on the base case results (data not shown).

#### 24 Weeks

**Fig. 4:**
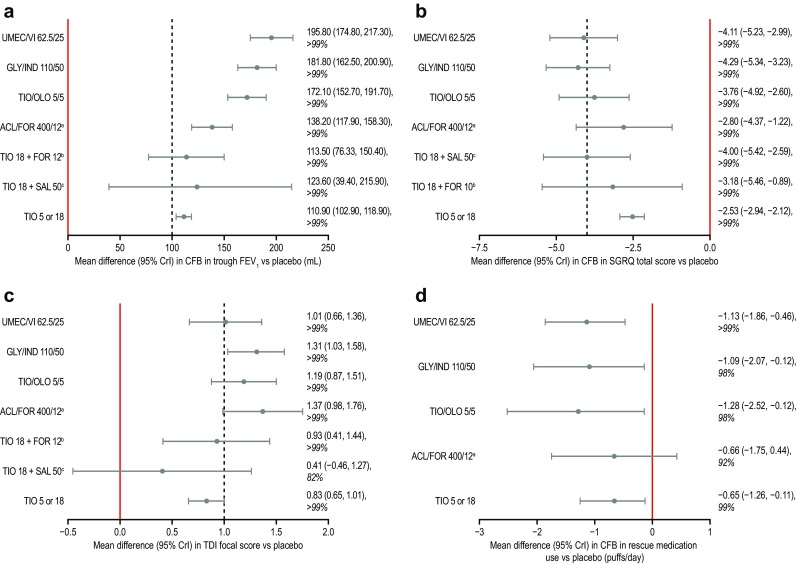
Forest plots showing differences for active treatments versus placebo in outcomes at 24 weeks. **a** CFB mean trough FEV_1_, **b** CFB mean SGRQ total score, **c** mean TDI focal score, **d** CFB mean rescue medication use (puffs/day). Results are reported as the mean with the 95% credible interval (Crl) in parenthesis, followed by the percentage probability of improved outcome versus placebo. *Black dotted lines* represent MCID values. ^*a*^Combined dose taken twice daily, ^*b*^FOR dose taken twice daily, ^*c*^SAL dose taken twice daily. All other doses are taken once daily. *ACL* aclidinium, *CFB* change from baseline, *CrI* credible interval, *FEV*_*1*_ forced expiratory volume in 1 second, *FOR* formoterol, *GLY* glycopyrronium, *IND* indacaterol, *MCID* minimal clinically important difference, *OLO* olodaterol, *SAL* salmeterol, *SGRQ* St George’s Respiratory Questionnaire, *TDI* transition dyspnea index, *TIO* tiotropium, *UMEC* umeclidinium, *VI* vilanterol

The base case analysis results at 24 weeks showed that all active therapies achieved improvements in trough FEV_1_ versus placebo greater than the 100 mL MCID with probabilities of improved outcomes of >99% (Table 1; Fig. 4a). In comparison with TIO, all FDCs demonstrated superiority, with probabilities of improved outcomes of >99% (ESM Table S7). However, the probabilities of improvement versus TIO for the open dual therapies [TIO 18 OD + salmeterol (SAL) 50 BID (TIO + SAL) and TIO 18 + FOR 12] were lower (61% and 56%, respectively).

Comparing the combination therapies, the OD FDCs (UMEC/VI, TIO/OLO, and GLY/IND 110/50) showed the greatest improvements versus placebo and had high probabilities (≥85%) of improved outcomes versus the other therapies (TIO + SAL, TIO 18 + FOR 12, and ACL/FOR). Between the OD FDCs, UMEC/VI had a high probability of improved outcomes versus TIO/OLO (96%; mean difference 23.74 mL; 95% CrI −3.31 to 50.73) and versus GLY/IND 110/50 (84%; mean difference 13.87 mL; 95% CrI −12.98 to 41.33) (ESM Table S7).

Results using data without imputed SEs were similar to the base case results, with only small differences (<5 mL) in changes in FEV_1_ for all comparisons (data not shown). Results of the scenario analysis (separating TIO 18 and TIO 5) were in line with the base case results, although the probability of improved outcomes with ACL/FOR versus TIO 5 was lower (75%; mean difference 12.57 mL; 95% CrI −22.83 to 49.43) than for the comparison with TIO 5 or 18 (>99%; mean difference 27.33 mL; 95% CrI 5.65 to 48.95).

### NMA Results: HRQoL Assessed Using SGRQ Total Score

#### 12 Weeks

The base case analysis results at 12 weeks showed that all active therapies achieved improvements in HRQoL, as shown by decreases in the SGRQ total score, versus placebo, with probabilities of improved outcomes of >99% (Table 1). These decreases were clinically relevant (greater than the MCID of 4 units [40]) for the combination therapies, with the exception of TIO 18 + FOR 12. In comparison with TIO, all therapies showed improvements in HRQoL, with decreases in the SGRQ score of <4 units and probabilities of improved outcomes of >99%, with the exception of TIO 18 + FOR 12 (probability of an improved outcome 75%; mean difference −1.01 units; 95% CrI −3.94 to 1.89) (ESM Table S8).

Comparing the combination therapies, the probabilities of improved outcomes using the FDCs (TIO/OLO, both doses of GLY/IND, and UMEC/VI) and the open dual combination TIO + OLO versus TIO 18 + FOR 12 were relatively high (≥68%). Reductions in the SGRQ score were similar between all FDCs and TIO + OLO, with the probabilities of improved outcomes for comparisons ranging from 38% to 57% (ESM Table S8).

Results with data without imputed SEs were generally similar to the base case results, with relatively small differences (<0.5 units) in changes in the SGRQ score for most comparisons except for those with GLY/IND 110/50, for which differences were greater (<1.1 units; data not shown). Comparisons with TIO 18 + FOR 12 were not included as these data were obtained by imputation.

#### 24 Weeks

The base case analysis results at 24 weeks showed that all active therapies achieved improvements in HRQoL, as shown by decreases in the SGRQ total score, versus placebo, with probabilities of improved outcomes of >99%; these decreases were clinically relevant (>4 units) for GLY/IND 110/50 and UMEC/VI only (Table 1; Fig. 4b). In comparison with TIO, all therapies showed improvements in HRQoL, with decreases in the SGRQ score of <4 units; the probability of improved outcomes was ≥98% for all therapies except for TIO 18 + FOR 10 (71%; mean difference −0.64 units; 95% CrI −2.93 to 1.64) and ACL/FOR (63%; mean difference −0.27 units; 95% CrI −1.89 to 1.36) (ESM Table S9).

Comparing the combination therapies, TIO + SAL and the OD FDCs (TIO/OLO, GLY/IND 110/50, and UMEC/VI) showed relatively high probabilities of improved outcomes versus ACL/FOR (≥83%) and TIO 18 + FOR 10 (≥68%). Improvements using TIO + SAL and the FDCs were similar, although relatively high probabilities of improved outcomes were shown versus TIO/OLO with GLY/IND 110/50 (76%; mean difference −0.53 units; 95% CrI −2.00 to 0.95) and UMEC/VI (67%; mean difference −0.35 units; 95% CrI −1.87 to 1.17).

Results with data without imputed SEs were generally similar to the base case results, with small differences (<0.2 units) in changes in the SGRQ score for most comparisons except those with ACL/FOR, for which differences were greater (<2.3 units; data not shown). With non-imputed data, the probabilities of improvement using ACL/FOR were considerably lower versus placebo (70%; mean difference −0.65 units; 95% CrI −3.07 to 1.80), TIO (8%; mean difference 1.78 units; 95% CrI −0.68 to 4.25) and all other combination therapies (<1–6%) than with imputed data. These differences are due to the inclusion of only a single trial reporting outcomes with ACL/FOR versus placebo in the non-imputed data (the ACLIFORM trial [41]) which reported a mean change from baseline in the SGRQ score of −0.65 units; however, the base case analysis also included the AUGMENT trial [42], which reported a considerably larger mean change from baseline of −4.36 units.

### NMA Results: Breathlessness Assessed by TDI Focal Score

#### 12 Weeks

The base case analysis results at 12 weeks showed that all active therapies achieved increases in TDI focal score versus placebo with probabilities of improved outcomes of ≥96% (Table 1). These increases were clinically relevant (greater than the MCID of 1.0 unit [43]) for the FDCs (TIO/OLO, both GLY/IND doses, and UMEC/VI) but not for TIO alone or TIO 18 + FOR 12. In comparison with TIO, the FDCs showed increases in the TDI focal score of <1.0 unit, with high probabilities of improved outcomes (≥96%), while the probability of an improved outcome with TIO 18 + FOR 12 was lower (56%; mean difference 0.07 units; 95% CrI −0.88 to 1.02) (ESM Table S10).

Comparing the combination therapies, the FDCs showed high probabilities of improved outcomes (≥77%) versus the only open dual therapy assessed (TIO 18 + FOR 12). TIO/OLO showed a high probability of improvement versus UMEC/VI (80%); the probability of improvement was also high versus UMEC/VI for GLY/IND 27.5/15.6 (86%) but was lower for GLY/IND 110/50 (61%) (ESM Table S10). No imputation analysis was necessary for these data.

#### 24 Weeks

The base case analysis results at 24 weeks showed that all active therapies achieved increases in TDI focal score versus placebo; the probability of improved outcomes was >99% for all therapies except TIO + SAL (82%; mean difference 0.41 units; 95% CrI −0.46 to 1.27) (Table 1; Fig. 4c). These improvements were clinically relevant (>1.0 unit) for the FDCs (TIO/OLO, GLY/IND 110/50, ACL/FOR, and UMEC/VI) but not for TIO alone or in open dual combination therapy. In comparison with TIO, the FDCs showed increases in TDI focal score of <1.0 unit, with high probabilities of improved outcomes (≥85%); the probabilities with open dual combinations were lower (65% for TIO 18 + FOR 12; 16% for TIO + SAL) (ESM Table S11).

Comparing the combination therapies, the FDCs and TIO 18 + FOR 12 all showed high probabilities (≥85%) of improved outcomes versus TIO + SAL. ACL/FOR and GLY/IND 110/50 showed high probabilities of improved outcomes versus TIO 18 + FOR 12 (91%; mean difference 0.44 units; 95% CrI −0.21 to 1.09; and 96%; mean difference: 0.38 units; 95% CrI: −0.06 to 0.82, respectively). ACL/FOR and GLY/IND 110/50 also showed high probabilities of improved outcomes versus UMEC/VI (91% and 92%, respectively) (ESM Table S11).

Results with data without imputed SEs were similar to the base case results, with only small differences (≤0.08 units) in changes in TDI score for all comparisons (data not shown). With non-imputed data, the probabilities of improvement using ACL/FOR were slightly lower versus TIO (94%; mean difference 0.48 units; 95% CrI −0.12 to 1.07) and all combination therapies (49–96%) than with imputed data.

### NMA Results: Puffs/day of Rescue Medication

#### 12 Weeks

The base case analysis results at 12 weeks showed that all active therapies achieved decreases in rescue medication use versus placebo, with probabilities of improved outcomes of ≥93% (Table 1). In comparison with TIO, combination therapies showed decreases in rescue medication use; the probabilities of improved outcomes were >99% for all therapies except TIO 18 + FOR 12 (78%; mean difference −0.25 puffs/day; 95% CrI −0.87 to 0.37) (ESM Table S12).

Comparing the combination therapies, TIO + IND and the FDCs (TIO/OLO, GLY/IND 27.5/15.6, and UMEC/VI) showed high probabilities of improved outcomes (≥82%) versus TIO 18 + FOR 12. TIO + IND showed a high probability of improvement versus TIO/OLO (88%), while both TIO + IND and GLY/IND 27.5/15.6 showed high probabilities of improvement versus UMEC/VI (98% and 94%, respectively) (ESM Table S12). However, a subsequent direct comparison of TIO + IND with UMEC/VI found no significant difference in rescue medication use [44].

Results with data without imputed SEs had no differences from the base case results (data not shown). Comparisons with TIO 18 + FOR 12 and TIO/OLO were not included as these data were obtained by imputation.

#### 24 Weeks

The base case analysis results at 24 weeks showed that all active therapies achieved decreases in rescue medication use versus placebo with probabilities of improved outcomes ≥92% (Table 1; Fig. 4d). In comparison with TIO, the OD FDCs (TIO/OLO, GLY/IND 110/50, and UMEC/VI) showed decreases in rescue medication use with high probabilities (≥87%) of improved outcomes; the probability of improvement with ACL/FOR was lower (51%; mean difference −0.01 puffs/day; 95% CrI −1.22 to 1.26).

Comparing the combination therapies, the OD FDCs showed relatively high probabilities of improved outcomes (≥78%) versus ACL/FOR. Reductions in rescue medication use were similar between the OD FDCs, with the probabilities of improved outcomes for comparisons ranging from 34% to 55% (ESM Table S13).

Results with data without imputed SEs were similar to the base case results, with small differences (≤0.2 puffs per day) in changes in rescue medication use for all comparisons. The probability of an improved outcome using GLY/IND 110/50 versus TIO was higher (>99%; mean difference −0.51 puffs/day; 95% CrI −0.91 to −0.10) than with imputed data. Comparisons with ACL/FOR and TIO/OLO were not included.

### Subgroup Analysis

The relative effects of different therapies on trough FEV_1_ at 12 and 24 weeks were assessed in subgroups of patients using and not using ICS, and in those with moderate disease or severe/very severe disease. Due to the limited data available for these subgroups, only some therapies could be compared. Differences in trough FEV_1_ at 24 weeks in favor of dual combinations [TIO 18 + FOR 12 and the FDCs (TIO/OLO, GLY/IND 110/50 and UMEC/VI)] versus placebo were greater for patients without ICS use (range 162–280 mL) than for patients with ICS use (range 118–180 mL; ESM Table S14) and for patients with moderate disease (range 147–276 mL) than patients with severe disease (58–119 mL; ESM Table S15). The probabilities of improved outcomes using TIO/OLO or UMEC/VI versus TIO 18 + FOR 12, and UMEC/VI versus TIO/OLO or GLY/IND 110/50, were higher in subgroups without ICS use and in those with moderate disease than in those with ICS use and with severe disease, respectively. However, the probabilities of improved outcomes using TIO 18 + FOR 12 versus TIO and GLY/IND 110/50 versus TIO/OLO were higher in subgroups with ICS use and with severe disease than in patients without ICS use or those with moderate disease, respectively.

## Discussion

In this study, the relative efficacy of LAMA + LABA combination bronchodilators was evaluated in patients with moderate-to-very-severe COPD using data from 44 RCTs on four outcomes of interest reported at 12 and/or 24 weeks after randomization: trough FEV_1_, SGRQ total score, TDI focal score, and daily rescue medication use. These outcomes were selected in accordance with the bulk of available data for RCTs involving dual bronchodilators.

Clinically relevant improvements were observed versus placebo over the 12- and/or 24-week time frame in trough FEV_1_ (improvements of ≥100 mL [39]), SGRQ total score (improvements of ≥4 units [40]), and TDI focal score (improvements of ≥1 unit [43]) with all FDC therapies, with the exception of TIO/OLO and ACL/FOR, both of which failed to show clinically relevant improvements in SGRQ total score at 24 weeks. Although no MCID has been established for rescue medication use, improvements were observed with all FDCs versus placebo at 12 and 24 weeks, with probabilities of improved outcomes of ≥92%. With open dual combinations of TIO with LABA therapies, improvements were observed versus placebo in all outcomes, but while improvements in trough FEV_1_ were clinically relevant, the magnitudes of treatment benefits for the PROs of SGRQ total score and TDI focal score were often below clinically relevant thresholds. These results are not surprising given the historical emphasis on improving lung function in the development of therapies for COPD [45]. The findings suggest that there may be a greater likelihood of a clinically important patient response with FDC bronchodilators compared with open dual regimens, particularly when comparing once- versus twice-daily treatments. To the best of our knowledge, comparative outcomes between FDCs and open dual combinations have not been assessed in other NMAs, and direct evidence in support of this claim is available from only one 6-month RCT that reported improvements in pre-bronchodilator FEV_1_ and forced vital capacity, and a significantly higher percentage of patients achieving a clinically relevant improvement in TDI, using GLY/IND 110/50 compared with TIO 18 + FOR 12 [46].

Once-daily FDCs showed consistent improvements versus TIO monotherapy in lung function, HRQoL, and rescue medication use after 12 and 24 weeks of treatment, with probabilities of better outcomes of ≥87% in all cases. However, less certainty of improvement was apparent with FDCs or open dual combinations containing FOR or SAL taken twice daily. When the TDI focal score was examined, improvements were observed with all FDCs versus TIO at 12 and 24 weeks, with probabilities of improvement of ≥85%, while the probability of improvement using open dual combinations was lower (16–65%). In contrast to improvements in trough FEV_1_ and SGRQ total score, no potential for efficacy differences emerged between once- or twice-daily regimens for the TDI assessments. The improvements observed using LAMA + LABA combination therapy compared with TIO monotherapy are consistent with results from several RCTs which directly compared various LAMA + LABA therapies with TIO [14–20]. This observation is therefore not unexpected and could be attributed to the direct agonist effect of the added LABA, as well as possible synergistic effects [13]. Some RCTs have reported improved lung function outcomes using LAMA + LABA therapies compared with LABA monotherapy; [15–17] these findings are consistent with the conclusions of two recent NMAs, which performed slightly different analyses to that presented in this study [47, 48].

It was notable that for most dual bronchodilators assessed at both time points, a greater magnitude of benefit in TDI focal score and SGRQ total score was observed at 12 compared with 24 weeks, but this diminution of response with time was not consistently observed for FEV_1_ and rescue medication use. This result may indicate that some PROs may be more subject to recall bias, as patients are required to recall their previous state in order to estimate changes [49], making them potentially less effective in discriminating between different active therapies over time, and suggests that shorter assessments may be more reliable for within-class comparisons. However, alternative approaches, such as comparing responder rates between two regimens, which has previously identified efficacy differences between dual bronchodilators, may be equally valid [46].

In this study, improvements in SGRQ total score and TDI focal score were never greater than the proposed MCIDs of 4 units [40] and 1 unit [43], respectively, when comparisons were made between active regimens. This finding highlights that dual combination therapy was associated with an incremental efficacy gain in PROs compared with standard of care monotherapy, a finding concordant with that reported in other meta-analyses comparing FDCs with their individual component LAMA or LABAs [47, 48]. By contrast, the magnitude of lung function benefit using FDCs compared with placebo was often twofold higher than that seen with TIO monotherapy. The current NMA identified a potential gradient of effectiveness emerging between the once-daily FDCs and those including twice-daily LABAs, particularly with regards to improvements in trough FEV_1_: at 12 weeks, GLY/IND and UMEC/VI demonstrated the greatest improvements, followed by TIO + IND, while improvements with ACL/FOR, TIO/OLO, TIO + OLO, and TIO 18 + FOR 10 were lower; at 24 weeks, UMEC/VI demonstrated the greatest improvement, followed by GLY/IND, then TIO/OLO, with ACL/FOR and the open dual combinations showing lower improvements. However, further trials are required to confirm these findings. Head-to-head trials of FDCs with results yet to be reported include two trials comparing GLY/IND with UMEC/VI (NCT02487446 and NCT02487498) and one trial comparing UMEC/VI with TIO/OLO (NCT02799784).

It is worth noting that the effectiveness of an inhaled therapy could potentially be affected by the delivery device used [50]. While several inhaler types are available, dry powder inhalers (DPIs) have become increasingly common for COPD therapies due to their multiple advantages over the more traditional pressurized metered-dose inhalers (pMDIs) [51]. However, different classes of DPIs are available, with ongoing debate about their relative advantages [51]. Of the therapies assessed in this NMA, most were delivered by either single-dose DPIs (TIO 18, IND, FOR 12, and the combined therapy GLY/IND) or multi-dose DPIs (FOR 10 and the combined therapies ACL/FOR and UMEC/VI). The exceptions were SAL, which was delivered by a pMDI, and TIO 5, OLO, and TIO/OLO, which were delivered by soft mist inhalers [52]. However, as the majority (40/44) of the studies included in the NMA were blinded, it is unlikely that these differences in inhaler type would be reflected in the results. Currently, while several studies have identified patient preferences between different inhaler types [53–55], there is limited evidence to demonstrate any differences in efficacy outcomes [56, 57]. Further research is therefore required to inform optimal device design, improve compliance, reduce handling errors and, ultimately, improve outcomes.

This NMA is an updated extension of our previous NMA comparing the efficacy of UMEC/VI with other dual bronchodilators, TIO, or placebo [28]. The previous review included 26 RCTs of ≥10 weeks’ duration, published in English up to April 2014. The expanded inclusion criteria in this update allowed the inclusion of a further 18 RCTs and enabled analysis of additional combination bronchodilators (TIO + OLO, TIO/OLO, GLY/IND 27.5/15.6, and ACL/FOR); also, TIO 5 was not included as a comparator in the previous analysis. However, the principal observation of similar efficacy between available combination bronchodilators with regards to PROs remained the same for both analyses. Improvements in trough FEV_1_ using different combination therapies were less similar; here, the magnitude of some of the differences between therapies were beyond accepted non-inferiority margins used for within-class comparisons (≥50 mL or ≥50% of the proposed MCID [8, 58]). Scenario analysis separating TIO 18 and TIO 5 showed that the addition of TIO 5 to the analysis had a negligible effect on measured outcomes. In this updated NMA we also performed subgroup analysis to examine potential confounding due to disease severity and concurrent therapy. The results highlight some additional differences in the magnitudes of improvements in lung function between therapies in patient subgroups with differing capacities for bronchodilation.

Two other recent NMAs have examined the comparative effects of available FDCs on lung function and PROs; neither included open dual combination therapies [48, 59]. The NMA by Schlueter et al. included 27 RCTs of ≥20 weeks’ duration, published up to September 2014, and assessed outcomes at 24 and 48 weeks [59]. RCTs were excluded based on concomitant treatments, and meta-regression rather than subgroup analysis was performed to assess the effects of baseline disease severity and concomitant ICS use on outcomes [59]. The NMA by Calzetta et al. included 22 RCTs of ≥3 months’ duration, published up to October 2015, and assessed outcomes for FDC LAMA/LABA combinations versus their component monotherapies when they were reported for each study; RCTs without comparisons of the dual therapy to a component monotherapy were excluded [48]. Despite the differences in inclusion criteria and analysis methods, the results obtained in both NMAs were consistent with those for the current analysis. Both NMAs assessed differences in SGRQ total score and TDI focal score using the percentage of responders, rather than the calculated differences in scores as in the present NMA; however, in accordance with our results, neither study reported any significant difference in these outcomes between FDCs [48, 59]. Both previous NMAs reported differences between the FDCs for trough FEV_1_. Schlueter et al. reported improvements with TIO/OLO, GLY/IND 110/50, and UMEC/VI above those seen with ACL/FOR that were of similar magnitudes to the improvements observed in the present study at the same time point (approximately 24 weeks) [59]. Calzetta et al. reported a similar potential gradient of effectiveness to that presented here, with UMEC/VI and GLY/IND 27.5/15.6 demonstrating the greatest improvements, followed by GLY/IND 110/50, TIO/OLO, then ACL/FOR [48].

A potential limitation of this and other NMAs of FDC bronchodilators is the low number of studies involving some treatments and the scarcity of direct comparisons of these with other active treatments. In particular, of the FDCs, only two studies presenting data for ACL/FOR were eligible for inclusion; both of these were ‘large’ trials (each with >300 patients receiving ACL/FOR [41, 42], considerably greater than the previously proposed cut-off for large trials of 100 patients [60]), thereby reducing the likelihood of any distortion of results due to a small study effect [61]. However, the heterogeneity of the studies included in this NMA was not assessed, so the extent of distortion is not known. Additionally, comparisons in this study were frequently calculated indirectly, so there is some uncertainty in the estimated efficacies. As with all meta-analyses, the potential influence of confounders constitutes a limitation. Due to the low number of studies involved, meta-regression to adjust for any confounders was infeasible, so subgroup analysis was performed. The greatest improvements in lung function were observed in non-ICS users and patients with moderate disease for all combination therapies versus placebo. However, it is still unclear whether any greater magnitude of difference in lung function between the therapies in these subgroups is likely to be matched by clinically important changes in PROs. Another limitation of this study is that the outcomes analyzed were restricted to those investigated in multiple RCTs of 12 or 24 weeks’ duration; other relevant endpoints that have been less commonly or inconsistently reported, such as exacerbation rate or time to first event in patients of varying exacerbation risk, were excluded.

## Conclusions

In an indirect treatment comparison using a Bayesian NMA, most but not all dual combination therapies showed improved outcomes versus placebo and versus TIO monotherapy at 12 and/or 24 weeks. The likelihood of improved outcomes appeared to be greater with FDCs compared with open dual combinations. The relative probabilities of improvement between available FDCs suggested broadly similar efficacy when assessed using the PROs of SGRQ total score, TDI focal score, and rescue medication use, while some differences between bronchodilators were observed for objective assessments using trough FEV_1_. While head-to-head RCTs would be required to provide robust evidence of efficacy differences between LAMA + LABA bronchodilators, indirect comparisons such as the present NMA allow the existing volume of RCT data to be employed to generate data which may be of use to healthcare payers and providers.

## Electronic supplementary material

Below is the link to the electronic supplementary material.

Supplementary material 1 (PDF 635 kb)
