# Supplementary material for: A Network Meta-Analysis of Long-Acting Muscarinic Antagonist (LAMA) and Long-Acting β2-Agonist (LABA) Combinations in COPD
Source: Pulm Ther. 2017 Aug 22;3(2):297–316. doi: 10.1007/s41030-017-0048-0 (PMC6964204; doi:10.1007/s41030-017-0048-0)
Supplement: Supplementary file 1 — Supplementary material 1 (PDF 635 kb) [file 41030_2017_48_MOESM1_ESM.pdf]

A Network Meta-Analysis of Long-Acting Muscarinic Antagonist (LAMA) and Long-Acting β_2-Agonist (LABA) Combinations in COPD – supplementary materials

Supplementary Table S1: Bibliographic Search Strategies

Table S1.1. Search strategy for the systematic review: MEDLINE and MEDLINE In-Process

**Database**	MEDLINE(R) In-Process & Other Non-Indexed Citations and MEDLINE(R)
**Platform**	Ovid
**Date of search**	2 Oct 2015
**Time limits**	1946 to 2015 week 39
**Filters**	Line 6 - 15 are from the search filter: Cochrane Highly Sensitive Search Strategy for identifying randomized trials in MEDLINE: sensitivity- and precision-maximizing version (2008 revision); Ovid format. Available from: http://handbook.cochrane.org/chapter_6/box_6.4.d_cochrane_hsss_2008_sensprec_ovid.htm (accessed 2 Oct 2015)

#	Searches	Results
1	(formoterol or eformoterol or foradil or oxis or atimos modulite or atock or perforomist or salmeterol or serevent or tiotropium or spiriva or Ba 679 BR or indacaterol or onbrez or arcapta or NVA-237 or NVA237 or (NVA adj "237") or glycopyrronium or glycopyrrolate or seebri or enurev breezhaler or aclidinium or tudorza pressair or eklira genuair or symbicort or advair or seretide or olodaterol or striverdi or umeclidinium or GSK573719 or vilanterol or GW642444 or QVA149 or relovair or zephyr or anoro ellipta). ti,ab,nm.	6157
2	exp Pulmonary Disease, Chronic Obstructive/ or exp Chronic obstructive lung disease/	40019
3	(COPD or chronic obstructive pulmonary disease or COAD or chronic obstructive airway disease or chronic obstructive lung disease or chronic bronchitis or emphysema).ti,ab.	67176
4	2 or 3	76919
5	1 and 4	1962
6	randomized controlled trial.pt.	411493
7	controlled clinical trial.pt.	91674
8	randomized.ab.	333334
9	placebo.ab.	168050
10	clinical trials as topic.sh.	178669
11	randomly.ab.	240412
12	trial.ti.	146789
13	or/6-12	997637
14	exp animals/ not humans.sh.	4116467

15	13 not 14	920230
16	5 and 15	985
17	limit 16 to (English or German)	933

ab, nm, pt, sh, ti: searches performed in abstract, name of substance, publication type, subject heading and title fields, respectively

Table S1.2 Search strategy for the systematic review: EMBASE

**Database**	EMBASE	
**Platform**	Ovid	
**Date of search**	2 Oct 2015	
**Time limits**	1988 to 2015 week 39	
**Filters**	Line 6 - 10 are from the search filter: Cochrane search terms used to identify EMBASE reports of randomized trials for inclusion in CENTRAL; Ovid format. Available from: http://handbook.cochrane.org/chapter_6/6_3_2_2_what_is_in_the_cochrane_central_register_of_controlled.htm (accessed 2 Oct 2015)	
**#**	**Searches**	**Results**
1	(formoterol or eformoterol or foradil or oxis or atimos modulite or atock or perforomist or salmeterol or serevent or tiotropium or spiriva or Ba 679 BR or indacaterol or onbrez or arcapta or NVA-237 or NVA237 or (NVA adj "237") or glycopyrronium or glycopyrrolate or seebri or enurev breezhaler or aclidinium or tudorza pressair or eklira genuair or symbicort or advair or seretide or olodaterol or striverdi or umeclidinium or GSK573719 or vilanterol or GW642444 or QVA149 or relovair or zephyr or anoro ellipta).ti,ab.	8350
2	exp Pulmonary Disease, Chronic Obstructive/ or exp Chronic obstructive lung disease/	75097
3	(COPD or chronic obstructive pulmonary disease or COAD or chronic obstructive airway disease or chronic obstructive lung disease or chronic bronchitis or emphysema).ti,ab.	82739
4	2 or 3	108091
5	1 and 4	3377
6	(random$ or factorial$ or crossover$ or cross over$ or cross-over$ or placebo$ or (doubl$ adj blind$) or (singl$ adj blind$) or assign$ or allocat$ or volunteer$).ti,ab.	1421388
7	crossover-procedure/ or double-blind procedure/ or randomized controlled trial/ or single-blind procedure/	412881
8	6 or 7	1498478
9	5 and 8	1700
10	limit 9 to (English or German)	1651

Table S1.3. Search strategy for the systematic review: Cochrane CENTRAL

**Database**	Cochrane CENTRAL	
**Platform**	Ovid	
**Date of search**	2 Oct 2015	
**Time limits**	1988 to 2015	
**Filters**	n.a.	
**#**	**Searches**	**Results**
1	(formoterol or eformoterol or foradil or oxis or atimos modulite or atock or perforomist or salmeterol or serevent or tiotropium or spiriva or Ba 679 BR or indacaterol or onbrez or arcapta or NVA-237 or NVA237 or (NVA adj "237") or glycopyrronium or glycopyrrolate or seebri or enurev breezhaler or aclidinium or tudorza pressair or eklira genuair or symbicort or advair or seretide or olodaterol or striverdi or umeclidinium or GSK573719 or vilanterol or GW642444 or QVA149 or relovair or zephyr or anoro ellipta).ti,ab,kw.	4995
2	Exp Pulmonary Disease, Chronic Obstructive/	2100
3	(COPD or chronic obstructive pulmonary disease or COAD or chronic obstructive airway disease or chronic obstructive lung disease or chronic bronchitis or emphysema).ti,ab,kw.	10562
4	1 and (2 or 3)	1810

ab, kw, ti: searches performed in abstract, keyword and title fields, respectively;

Table S1.4. Search strategy for the systematic review: Cochrane CDSR

**Database**	Cochrane CDSR	
**Platform**	Ovid	
**Date of search**	2 Oct 2015	
**Time limits**	1988 to 2014	
**Filters**	n.a.	
**#**	**Searches**	**Results**
1	(formoterol or eformoterol or foradil or oxis or atimos modulite or atock or perforomist or salmeterol or serevent or tiotropium or spiriva or Ba 679 BR or indacaterol or onbrez or arcapta or NVA-237 or NVA237 or (NVA near/3 237) or glycopyrronium bromide or glycopyrrolate or seebri or enurev breezhaler or aclidinium bromide or tudorza pressair or eklira genuair or symbicort or advair or seretide or olodaterol or striverdi or umeclidinium or GSK573719 or vilanterol or GW642444 or QVA149 or relovair or zephyr or anoro ellipta).ti,ab,kw.	47
2	(COPD or chronic obstructive pulmonary disease or COAD or chronic obstructive airway disease or chronic obstructive lung disease or chronic bronchitis or emphysema).ti,ab,kw.	131
3	1 and 2	23

ab, kw, ti: searches performed in abstract, keyword and title fields, respectively;

Table S1.5 Search strategy for the systematic review: DARE Database

**Database**	DARE	
**Platform**	Ovid	
**Date of search**	2 Oct 2015	
**Time limits**	No time limits	
**Filters**	n.a.	
**#**	**Searches**	**Results**
1	(formoterol or eformoterol or foradil or oxis or atimos modulite or atock or perforomist or salmeterol or serevent or tiotropium or spiriva or Ba 679 BR or indacaterol or onbrez or arcapta or NVA-237 or NVA237 or (NVA and "237") or glycopyrronium bromide or glycopyrrolate or seebri or enurev breezhaler or aclidinium bromide or tudorza pressair or eklira genuair or symbicort or advair or seretide or olodaterol or striverdi or umeclidinium or GSK573719 or vilanterol or GW642444 or QVA149 or relovair or zephyr or anoro ellipta).ti,kw,ft.	82
2	(COPD or chronic obstructive pulmonary disease or COAD or chronic obstructive airway disease or chronic obstructive lung disease or chronic bronchitis or emphysema).ti,kw,ft	397
3	1 and 2	47

ti, kw, ft: searches performed in title, keyword and full text, respectively.

Table S1.6. Search strategy for the systematic review: Health Technology Assessment Database

**Database**	HTA	
**Platform**	Ovid	
**Date of search**	2 Oct 2015	
**Time limits**	No time limits	
**Filters**	n.a.	
**#**	**Searches**	**Results**
1	(formoterol or eformoterol or foradil or oxis or atimos modulite or atock or perforomist or salmeterol or serevent or tiotropium or spiriva or Ba 679 BR or indacaterol or onbrez or arcapta or NVA-237 or NVA237 or (NVA and "237") or glycopyrronium bromide or glycopyrrolate or seebri or enurev breezhaler or aclidinium bromide or tudorza pressair or eklira genuair or symbicort or advair or seretide or olodaterol or striverdi or umeclidinium or GSK573719 or vilanterol or GW642444 or QVA149 or relovair or zephyr or anoro ellipta).mp.	33
2	(COPD or chronic obstructive pulmonary disease or COAD or chronic obstructive airway disease or chronic obstructive lung disease or chronic bronchitis or emphysema).mp.	145
3	Exp Pulmonary Disease, Chronic Obstructive/	118
4	1 and (2 or 3)	29

mp: searches performed in any field.

Supplementary Table S2: Registry Search Strategies

Table S2.1 Search strategy for Clinicaltrials.gov

**Trial registry**	clinicaltrials.gov
**URL**	http://www.clinicaltrials.gov/
**Date of search**	2 Oct 2015
**Search strategy**	COPD OR COAD OR "Chronic obstructive pulmonary disease" OR "Chronic obstructive lung disease" OR "chronic obstructive airway disease" OR "chronic bronchitis" OR "emphysema" | Phase 2, 3, 4
**Results**	1252

Table S2.2. Search strategy for World Health Organization International Clinical Trials Registry Platform

**Trial registry**	WHO International Clinical Trials Registry Platform (ICTRP)
**URL**	http://apps.who.int/trialsearch/AdvSearch.aspx
**Date of search**	2 Oct 2015
**Search strategy**	COPD OR chronic obstructive pulmonary disease OR COAD OR chronic obstructive airway disease OR chronic obstructive lung disease OR chronic bronchitis OR emphysema
**Results**	3689

*The WHO ICTRP imports records from several registries. Trials are sometimes recorded in more than one registry. These records can refer to each other using a secondary identification number. The search portal uses this secondary identification number to group records about the same trial together in the search results.

All results were reported in an excel database. However, WHO ICTRP also collects data from Asian registries. As non-Caucasian population is an exclusion criterion, trials listed on national non-Caucasian registries were excluded for population not of interest. (i.e. Chinese Clinical Trial Registry; Clinical Trials Registry (ChiCTR) – India (CTRI); Iranian Registry of Clinical Trials; Japan Primary Registries Network).

Table S2.3. Search strategy for Current Controlled Trials

**Trial registry**	Current controlled trials
**URL**	http://www.controlled-trials.com/editAdvancedSearch
**Date of search**	8 Oct 2015
**Search strategy**	(Chronic obstructive pulmonary disease) in Condition
**Results**	159

ClinicalTrials.gov was removed from the list of resources searched in this aggregated database, as clinicaltrials.gov was searched directly in a separate search.

Table S2.4. Search strategy for EU Clinical Trials Register

**Trial registry**	EU Clinical Trials Register (EU-CTR)
**URL**	www.clinicaltrialsregister.eu
**Date of search**	8 Oct 2015
**Search strategy**	(COPD OR chronic obstructive pulmonary disease OR COAD OR chronic obstructive airway disease OR chronic obstructive lung disease OR chronic bronchitis OR emphysema) AND (Phase II OR Phase III or Phase IV [Select trial phase])
**Results**	354

Table S2.5. Search strategy for Klinische Prüfungen PharmNet.Bund

**Trial registry**	Klinische Prüfungen PharmNet.Bund
**URL**	http://www.pharmnet-bund.de/dynamic/de/klinische-pruefungen/index.htm
**Date of search**	8 Oct 2015
**Search strategy**	COPD in Textfelder
**Results**	201

*Table S2.6. Search strategy for International Prospective Register of Systematic Reviews**

**Trial registry**	International Prospective Register of Systematic Reviews (PROSPERO)
**URL**	http://www.crd.york.ac.uk/prospero/search.asp
**Date of search**	8 Oct 2015
**Search strategy**	Separate searches for: COPD [ALL FIELDS] or chronic obstructive pulmonary disease [ALL FIELDS] or COAD [ALL FIELDS] or chronic obstructive airway disease [ALL FIELDS] or chronic obstructive lung disease [ALL FIELDS]
**Results**	166

*Please note that each search term has to be searched for manually, and then any duplicates removed at the end.

Table S2.7. Search strategy for NIHR Health Technology Assessment

**Trial registry**	National Institute for Health Research - Health Technology Assessment (NIHR HTA)
**URL**	http://www.nets.nihr.ac.uk/projects
**Date of search**	8 Oct 2015
**Search strategy**	COPD [Keywords] and HTA [programme] in the advanced search
**Results**	17

Supplementary Table S3: Key characteristics of all studies included in the NMA (arms of interest only)

Study	Treatment	Trial duration	Inclusion criteria	Background treatment
**DB2113373[1-3] (NCT01313650)**	UMEC/VI 62.5/25 mcg OD<br><br>Placebo	24 weeks	Outpatient; ≥40 years of age; smoking history ≥10 pack-years; diagnosed with COPD; post-salbutamol FEV_1/FVC ratio of <0.70 and a post-salbutamol FEV_1 of ≤70%	Allowed: ICS at a dose of up to 1000 mcg/day of FP or equivalent, salbutamol/albuterol as rescue<br><br>Not allowed: LABAs, ICS/LABA combinations, SABAs, SAMAs, ICS/SABA combinations
**DB2113374[4-6] (NCT01316913)**	UMEC/VI 62.5/25 mcg OD<br><br>UMEC/VI 125/25 mcg OD<br><br>TIO 18 mcg OD	24 weeks	Outpatient; ≥40 years of age; smoking history ≥10 pack-years; diagnosed with COPD; post-salbutamol FEV_1/FVC ratio of <0.70 and a post-salbutamol FEV_1 of ≤70%	Allowed: ICS at a dose of up to 1000 mcg/day of FP or equivalent, salbutamol/albuterol as rescue<br><br>Not allowed: oral LABAs, oral and inhaled SABAs, inhaled SAMAs, ICS/SABA combinations
**DB2113360[7-9] (NCT01316900)**	UMEC/VI 62.5/25 mcg OD<br><br>UMEC/VI 125/25 mcg OD<br><br>TIO 18 mcg OD	24 weeks	Outpatient; ≥40 years of age; smoking history ≥10 pack-years; diagnosed with COPD, post-salbutamol FEV_1 ≤70% and post-salbutamol FEV_1/FVC ratio <0.7	Allowed: ICS at a dose of up to 1000 mcg/day of FP or equivalent, salbutamol/albuterol as rescue<br><br>Not allowed: LABAs, SABAs, SAMAs, ICS/SABA combinations
**ZEP117115[10-12] (NCT01777334)**	UMEC/VI 62.5/25 mcg OD<br><br>TIO 18 mcg OD	24 weeks	Outpatient; ≥40 years of age; smoking history ≥10 pack-years; diagnosed with COPD; post-salbutamol FEV_1/FVC ratio of <0.70 and a post-salbutamol FEV_1 of ≤70%	Allowed: ICS at a dose of up to 1000 mcg/day of FP or equivalent, salbutamol/albuterol as rescue<br><br>Not allowed: oral LABAs, oral and inhaled SABAs, inhaled SAMAs, ICS/LABA combinations, ICS/SABA combinations

Study	Treatment	Trial duration	Inclusion criteria	Background treatment
**Aaron 2007[13, 14]**	TIO 18 mcg OD + SAL 25 mcg 2 puffs BID<br><br>TIO 18 mcg OD + placebo 2 puffs BID	52 weeks	≥35 years of age; smoking history ≥10 pack years; diagnosis of moderate or severe COPD; ≥1 COPD exacerbation requiring systemic steroids or antibiotics in previous 12 months; post-bronchodilator FEV_1 ≤65%; FEV_1/FVC <70%	Allowed: albuterol for relief of symptoms<br><br>Not allowed: ICS, LABA, anticholinergics
**Abrahams 2013 (NCT00528996)[15]**	TIO 5 mcg OD<br><br>Placebo	24 weeks	≥40 years of age; current or ex-smokers with a smoking history of >10 pack-years; COPD with a FEV_1 (30 min post-salbutamol/albuterol) <80% of predicted normal; FEV_1/FVC ≤70%	Allowed: rescue salbutamol, antibiotics, oral corticosteroids (≤10 mg daily); inhaled LABAs, oral ICS, theophylline preparations and mucolytic agents not containing bronchodilators. Short-acting anticholinergic drugs were allowed during the 2-week baseline period and the 3-week follow-up period<br><br>Not allowed: SABAs other salbutamol, anticholinergics other than the study drug and their combinations
**ACLIFORM COPD (NCT01462942; Singh 2014)[16]**	ACL/FOR 400/12 mcg BID<br><br>Placebo	24 weeks	≥40 years of age; smoking history of ≥10 pack-years; FEV_1 <80% and ≥30% predicted; post-bronchodilator FEV_1/FVC <70%	Allowed: rescue use of albuterol/salbutamol; other COPD medications, such as theophylline, ICS, oral or parenteral corticosteroids (≤10 mg/day or 20 mg every other day of prednisone) were allowed if treatment was stable ≥4 weeks prior to screening.<br><br>Not allowed: LABAs other than investigational treatment
**ANHELTO 1 (NCT01694771; ZuWallack 2014)[17]**	TIO 18 mcg OD + OLO 5 mcg OD<br><br>TIO 18 mcg OD	12 weeks	≥40 years of age; smoking history of >10 pack-years; post-bronchodilator FEV_1 ≥30% and <80% of predicted normal; post-bronchodilator FEV_1/FVC <70% (GOLD stage 2–3)	Allowed: ICS, oral (≤10 mg prednisone per day, or equivalent) and injected steroids, cromolyn sodium/nedocromil sodium, antihistamines, antileukotrienes, methylxanthines, mucolytics,

Study	Treatment	Trial duration	Inclusion criteria	Background treatment
				and theophyllines. Albuterol permitted as rescue medication only.<br><br>Not allowed: concurrent ICS in fixed combination with LABA; ICS/SABA combinations; SAMA/SABA combinations or phosphodiesterase type 4 inhibitors.
**ANHELTO 2 (NCT01696058; ZuWallack 2014)[17]**	TIO 18 mcg OD + OLO 5 mcg OD<br><br>TIO 18 mcg OD	12 weeks	≥40 years of age; smoking history of >10 pack-years; post-bronchodilator FEV_1 ≥30% and <80% of predicted normal; post-bronchodilator FEV_1/FVC <70% (GOLD stage 2–3)	Allowed: ICS, oral (≤10 mg prednisone per day, or equivalent) and injected steroids, cromolyn sodium/nedocromil sodium, antihistamines, antileukotrienes, methylxanthines, mucolytics, and theophyllines. Albuterol permitted as rescue medication only.<br><br>Not allowed: concurrent ICS in fixed combination with LABA, ICS/SABA combinations, SAMA/SABA combinations or phosphodiesterase Type 4 inhibitors.
**AUGMENT COPD (NCT01437397; D'Urzo 2014)[18]**	ACL/FOR 400/12 mcg BID<br><br>Placebo	24 weeks	≥40 years of age; smoking history ≥10 pack-years; FEV_1 <80% and ≥30% predicted; post-bronchodilator FEV_1/FVC <70%	Allowed: rescue use of albuterol/salbutamol; other COPD medications such as theophylline, ICS, oral or parenteral corticosteroids (≤10 mg/day or 20 mg every other day of prednisone) were allowed if treatment was stable ≥4 weeks prior to screening.<br><br>Not allowed: LABAs other than investigational treatment

Study	Treatment	Trial duration	Inclusion criteria	Background treatment
**Bateman 2010a (NCT00387088)[19]**	TIO 5 mcg OD<br><br>Placebo	48 weeks	≥40 years of age; smoking history of ≥10 pack-years; pre-bronchodilator FEV_1 ≤60% of predicted normal; FEV_1/FVC ≤70%	At the screening and baseline visits, and the visits at Weeks 4, 24 and 48, administration of concomitant medications before pulmonary function tests was restricted to avoid interference with the tests. The restricted period was 8 h for SABAs, 12 h for any LABA- or ICS-containing medication, and 24–48 h for xanthines, depending on the drug release kinetics of the formulation.
**Bateman 2010b (NCT00168844, BI study number 205.254; NCT00168831, BI study number 205.255)[20]**	TIO 5 mcg OD<br><br>Placebo	48 weeks	≥40 years of age; smoking history of ≥10 pack-years; pre-bronchodilator FEV_1 ≤60% predicted; FEV_1/FVC ≤70%	Allowed: oral and inhaled corticosteroids, theophylline preparations, mucolytic agents and antileukotrienes, if stabilized for at least 6 weeks prior to and during the study. Salbutamol MDI was used as rescue medication.<br><br>Patients on LABA and ICS were switched to ICS monotherapy prior to run-in.
**Beeh 2006[21]**	TIO 18 mcg OD<br><br>Placebo	12 weeks	≥40 years of age; smoking history of ≥10 pack-years; stable COPD; FEV_1 ≤70% predicted; FEV_1/FVC <70%	Not allowed: LABAs and LAMAs/SAMAs other than the investigational therapy were stopped at the beginning of the study.<br><br>SABAs were uniformly replaced by fenoterol MDI for use as needed.
**BI trial number 205.269 (NCT00144326)[22]**	TIO 18 mcg OD<br><br>Placebo	12 weeks	≥40 years of age; smoking history of ≥10 pack-years; FEV_1 <60% predicted; FEV_1/FVC <70%	NR

Study	Treatment	Trial duration	Inclusion criteria	Background treatment
**Brusasco 2003[23]**	TIO 18 mcg OD<br>Placebo	24 weeks	>40 years of age; smoking history of >10 pack-years; FEV_1 ≤65% predicted; FEV_1/FVC ≤70%	NR
**Casaburi 2000[24]**	TIO 18 mcg OD<br>Placebo	13 weeks	≥40 years of age; smoking history of >10 pack-years; diagnosis of COPD as defined by ATS; FEV_1 ≤65% predicted; FEV_1/FVC ≤70%	Allowed: stable doses of theophylline, ICS, oral prednisone<br>Not allowed: other inhaled or oral bronchodilators
**Casaburi 2002[25]**	TIO 18 mcg OD<br>Placebo	56 weeks	≥40 years of age; smoking history of ≥10 pack years; FEV_1 ≤65% predicted; FEV_1/FVC ≤70%	Allowed: stable doses of theophylline, ICS, oral prednisone
**Chan 2007 (Boehringer Ingelheim [BI] trial number 205.259)[26]**	TIO 18 mcg OD<br>Placebo	48 weeks	≥40 years of age; smoking history of ≥10 pack-years; FEV_1 ≤65% predicted; FEV_1/FVC ≤70%; ≥1 exacerbation in the previous year excluding the 6 weeks prior to start (later amended to ≥1 exacerbation in the previous 2 years)	Allowed: stable dose oral corticosteroids, ICS, theophylline preparations, mucolytic preparations (not containing bronchodilators), LABAs
**Covelli 2005[27]**	TIO 18 mcg OD<br>Placebo	12 weeks	FEV_1 ≤60% predicted; FEV_1/FVC ≤70%. Patients were excluded if they experienced an exacerbation within 6 weeks prior to start	Allowed: ICS, LABAs and theophyllines<br>Not allowed: cromones, leukotriene antagonists, inhaled anticholinergics
**Donohue 2002[28]**	TIO 18 mcg OD<br>Placebo	24 weeks	≥40 years of age; smoking history of >10 pack-years; FEV_1 ≤60% predicted; FEV_1/FVC ≤70%	Allowed: usual ICS and oral steroids<br>Not allowed: inhaled anticholinergic LABAs

Study	Treatment	Trial duration	Inclusion criteria	Background treatment
**Donohue 2010[29]**	TIO 18 mcg OD<br>Placebo	26 weeks	≥40 years of age; smoking history of ≥20 pack-years; diagnosis of moderate-to-severe COPD (GOLD criteria); post-bronchodilator (within 30 min of inhaling albuterol 360 mcg) FEV_1 <80% and ≥30% predicted; FEV_1/FVC <70%	Allowed: albuterol as needed; continued ICS monotherapy if stable for 1 month before screening (dose and regimen were to remain stable throughout the study)<br>Not allowed: LAMAs or LABAs other than the investigational therapy (discontinued prior to start with appropriate washout); patients receiving ICS/LABA combinations were switched to ICS monotherapy at an equivalent dose.
**ENLIGHTEN (Dahl 2013, NCT01120717)[30-32]**	GLY/IND 110/50 mcg OD<br>Placebo	52 weeks	≥40 years of age; smoking history of ≥10 pack years; diagnosis of moderate or severe COPD (stage II or III according to GOLD 2008 criteria); post-bronchodilator FEV_1 <80% and ≥30% predicted; post-bronchodilator FEV_1/FVC <0.70	Allowed: Albuterol as rescue medication, ICS monotherapy<br>Not allowed: long-acting bronchodilators (LABA, LAMA, theophylline), SAMAs
**FLIGHT1 (NCT01727141; Mahler 2015)[33]**	GLY/IND 27.5/15.6 mcg BID<br>Placebo	12 weeks	≥40 years of age; smoking history of ≥10 pack years; stable COPD according to GOLD 2011 guidelines; post-bronchodilator FEV_1 ≥30% and <80% predicted; post-bronchodilator FEV_1/FVC <0.70 at run-in; mMRC grade ≥2 at run-in	Allowed: background ICS<br>Not allowed: COPD-related medications other than investigational therapy (LAMAs, SAMAs, ICS/LABA combinations, SABA, SAMA/SABA combinations, etc.)<br>Patients receiving SSRIs, ICS, intranasal steroids, H_1-antagonists, or inactivated influenza, pneumococcal or any other inactivated vaccines should be excluded unless on stable dose.
**FLIGHT2 (NCT01712516; Mahler 2015)[33]**	GLY/IND 27.5/15.6 mcg BID<br>Placebo	12 weeks	≥40 years of age; smoking history of ≥10 pack years; stable COPD according to GOLD 2011 guidelines; post-bronchodilator FEV_1 ≥30% and	Allowed: background ICS<br>Not allowed: COPD-related medications other than investigational therapy (LAMAs, SAMAs,

Study	Treatment	Trial duration	Inclusion criteria	Background treatment
			<80% predicted; post-bronchodilator FEV_1/FVC <0.70 at run-in; mMRC grade ≥2 at run-in	ICS/LABA combinations, SABA, SAMA/SABA combinations, etc.)<br><br>Patients receiving SSRIs, ICS, intranasal steroids, H_1-antagonists, or inactivated influenza, pneumococcal or any other inactivated vaccine should be excluded unless on stable dose.
**GLOW2 (Kerwin 2012, NCT00929110)[34]**	TIO 18 mcg OD<br><br>Placebo	52 weeks	≥40 years of age; smoking history of ≥10 pack-years; diagnosis of moderate-to-severe stable COPD; post-bronchodilator FEV_1 ≥30% and <80% predicted; post-bronchodilator FEV_1/FVC <0.70	Allowed: inhaled or intranasal corticosteroids and H_1-antagonists; rescue salbutamol/albuterol<br><br>Not allowed: LAMAs (min 7 days before run-in); LABAs or ICS/LABA combinations (min 48 h before run-in).
**INTRUST-1 (Mahler 2012, NCT00846586)[35-37]**	TIO 18 mcg OD + IND 150 mcg OD<br><br>TIO 18 mcg OD	12 weeks	≥40 years of age; smoking history of ≥10 pack-years; post-bronchodilator FEV_1 ≤65% and ≥30% predicted; post-bronchodilator FEV_1/FVC <70%	Allowed: ICS monotherapy, rescue salbutamol/albuterol<br><br>Not allowed: LABAs, SABAs other than the investigational therapy, theophylline, anticholinergics
**INTRUST-2 (Mahler 2012, NCT00877383)[35, 38, 39]**	TIO 18 mcg OD + IND 150 mcg OD<br><br>TIO 18 mcg OD	12 weeks	≥40 years of age; smoking history of ≥10 pack-years; post-bronchodilator FEV_1 ≤65% and ≥30% predicted; post-bronchodilator FEV_1/FVC <70%	Allowed: ICS monotherapy, rescue salbutamol/albuterol<br><br>Not allowed: LABAs, SABAs other than the investigational therapy, theophylline, anticholinergics

Study	Treatment	Trial duration	Inclusion criteria	Background treatment
**Moita 2008[40]**	TIO 18 mcg OD<br>Placebo	12 weeks	FEV_1 ≤70% predicted; FEV_1/FVC ≤70%. Patients with ≥3 exacerbations in the previous year or an exacerbation within 6 weeks prior to start were excluded.	Allowed: LABAs, theophylline, mucolytics, ICS, stable doses oral corticosteroids; temporary increases in theophylline or oral steroids for exacerbations.<br>Not allowed: theophylline 24 h preparation
**Niewoehner 2005[41]**	TIO 18 mcg OD<br>Placebo	6 months	≥40 years of age; smoking history of ≥10 pack-years; FEV_1 ≤60% predicted; FEV_1/FVC ≤70%. Patients were excluded if not recovered from exacerbation ≥30 days prior to start.	Allowed: all other respiratory medications (including ICS and LABAs)<br>Not allowed: open-label anticholinergic bronchodilator
**OTEMTO 1 (NCT01964352; Singh 2015)[42]**	TIO/OLO 5/5 mcg OD<br>TIO 5 mcg OD<br>Placebo	12 weeks	≥40 years of age; smoking history of >10 pack-years; moderate-to-severe COPD (GOLD stage 2–3); post-bronchodilator FEV_1 ≥30% and <80% predicted; FEV_1/FVC <70% predicted	Allowed: open-label rescue salbutamol; continued ICS therapy if patients were on a stable dose for 6 weeks prior to screening.<br>Not allowed: LAMAs or LABAs other than study medication.<br>SAMAs were permitted only during the screening period.
**OTEMTO 2 (NCT02006732; Singh 2015)[42]**	TIO/OLO 5/5 mcg OD<br>TIO 5 mcg OD<br>Placebo	12 weeks	≥40 years of age; smoking history of >10 pack-years; moderate-to-severe COPD (GOLD stage 2–3); post-bronchodilator FEV1 ≥30% and <80% predicted; FEV_1/FVC <70% predicted	Allowed: open-label rescue salbutamol; continued ICS therapy if patients were on a stable dose for 6 weeks prior to screening.<br>Not allowed: LAMAs or LABAs other than study medication.<br>SAMAs were permitted only during the screening period.

Study	Treatment	Trial duration	Inclusion criteria	Background treatment
**QUANTIFY (NCT01574651; Buhl 2015)[43]**	GLY/IND 110/50 mcg OD<br>TIO 18 mcg OD + FOR 12 mcg BID	26 weeks	≥40 years of age; smoking history of ≥10 pack-years; moderate-to-severe stable COPD (GOLD stage 2–3); post-bronchodilator FEV_1 ≥30% and <80% predicted; post-bronchodilator FEV_1/FVC <0.7	Allowed: rescue salbutamol; SSRIs; H_1-antagonists (except mizolastine or terfenadine), inactivated influenza, pneumococcal or any other inactivated vaccine if not administered within 48 h before the study visit. Patients receiving ICS at baseline continued treatment at the same or equivalent dose and regimen.
**SHINE (Bateman 2013, NCT01202188)[44-46]**	GLY/IND 110/50 mcg OD<br>TIO 18 mcg OD<br>Placebo	26 weeks	≥40 years of age; smoking history of ≥10 pack-years; diagnosis of moderate or severe COPD (stage II or III according to GOLD 2008 criteria); post-bronchodilator FEV_1 <80% and ≥30% predicted; post-bronchodilator FEV_1/FVC <0.70	Allowed: salbutamol/albuterol as rescue medication, inhaled or intranasal corticosteroids in constant doses<br>Not allowed: LABA, LAMA, LABA/ICS combinations
**SPARK (Wedzicha 2013, NCT01120691)[47-49]**	GLY/IND 110/50 mcg OD<br>TIO 18 mcg OD	64 weeks	≥40 years of age; smoking history of ≥10 pack-years; diagnosis of severe or very severe COPD (stage III or IV according to GOLD 2008 criteria); post-bronchodilator FEV_1 <50% predicted; FEV_1/FVC <0.70; ≥1 exacerbation in the previous 12 months requiring systemic corticosteroids or antibiotics	Allowed: salbutamol, stable ICS doses<br>Not allowed: long-acting bronchodilators other than investigational therapies
**Tashkin 2009[50]**	TIO 18 mcg OD + FOR 12 mcg BID<br>TIO 18 mcg OD + Placebo BID	12 weeks	≥40 years of age; post-bronchodilator FEV_1 <70% and >30% predicted or >0.75 L, whichever was lower, at run-in; FEV_1/FVC <0.70	Allowed: rescue albuterol; continued use of prior stable ICS regimens and systemic corticosteroids for treatment of exacerbations

Study	Treatment	Trial duration	Inclusion criteria	Background treatment
**TIOSPIR (NCT01126437; Wise 2013; Anzueto 2015) [51, 52]**	TIO 18 mcg OD<br>TIO 5 mcg OD	2.3 years	≥40 years of age; smoking history of ≥10 pack-years; diagnosis of COPD with a post-bronchodilator FEV_1 ≤70% predicted and post-bronchodilator FEV_1/FVC ≤70%	Allowed: all COPD medications except other inhaled anticholinergic agents
**TIPHON (Tonnel 2008, NCT00274053)[53]**	TIO 18 mcg OD<br>Placebo	36 weeks	≥40 years of age; smoking history of >10 pack-years; FEV_1 20-70% predicted; FEV_1/SVC ≤70%	Allowed: stable doses of theophylline preparations (excluding 24-h preparations), mucolytics, ICS and oral steroids
**TONADO-1 (NCT01431274; Buhl 2015; Ferguson 2015) [54, 55]**	TIO/OLO 5/5 mcg OD<br>TIO 5 mcg OD	52 weeks	Outpatient; ≥40 years of age; smoking history of >10 pack-years; moderate-to-very-severe COPD (GOLD stage 2–4); post-bronchodilator FEV_1 <80% predicted; post-bronchodilator FEV_1/FVC <70%	Allowed: background ICS
**TONADO-2 (NCT01431287; Buhl 2015; Ferguson 2015)[54, 55]**	TIO/OLO 5/5 mcg OD<br>TIO 5 mcg OD	52 weeks	Outpatient; ≥40 years of age; smoking history of >10 pack-years; moderate-to-very-severe COPD (GOLD stage 2–4); post-bronchodilator FEV_1 <80% predicted; post-bronchodilator FEV_1/FVC <70%	Allowed: background ICS
**Troosters 2014 (NCT00523991)[56]**	TIO 18 mcg OD<br>Placebo	24 weeks	≥40 and ≤80 years of age; smoking history of ≥10 pack-years; diagnosis of COPD (GOLD stage 2): post-bronchodilator FEV_1/FVC <0.7; FEV_1 ≥50% and <80% predicted; MRC dyspnea score ≥2	Allowed: open-label rescue salbutamol as needed; oral corticosteroid treatment for ≤2 weeks for acute exacerbations<br>Not allowed (during the 6 months before and throughout the study): LABAs; SABAs (except salbutamol after visit 1); oral β_2-agonists; ICS; ICS/LABA; oral corticosteroids; theophylline; leukotriene antagonists; all open-label

Study	Treatment	Trial duration	Inclusion criteria	Background treatment
				anticholinergics (including ipratropium, TIO, combinations of these and oxitropium)
**UPLIFT (Tashkin 2008, 2012 and 2014; Celli 2009; Buhl 2012)[57-61]**	TIO 18 mcg OD<br>Placebo	4 years	≥40 years of age; smoking history of >10 pack-years; FEV_1 ≤70% predicted; FEV_1/FVC ≤70%. Patients with exacerbations within 4 weeks prior to start were excluded.	Allowed: all respiratory medications except other inhaled anticholinergics
**Verkindre 2006[62]**	TIO 18 mcg OD<br>Placebo	12 weeks	FEV_1 ≤50% predicted; FEV_1/SVC ≤70%; residual volume ≥125%. Patients on unstable doses of oral corticosteroids within 6 weeks prior to start were excluded.	Allowed: stable doses of oral corticosteroids, ICS, theophylline preparations, mucolytic agents<br>Not allowed: SABAs, oral β_2-agonists, LABAs
**Vogelmeier 2008 (NCT00134979)[63]**	TIO 18 mcg OD + FOR 10 mcg BID<br>TIO 18 mcg OD<br>Placebo	24 weeks	≥40 years of age at onset of COPD; smoking history of ≥10 pack-years; FEV_1<70% predicted and ≥1.00 L; FEV_1/FVC <70%	Allowed: salbutamol, ICS monotherapy
**Voshaar 2008 (NCT00239473, BI study number 205.251; NCT00240435, BI study number 205.252)[64]**	TIO 5 mcg OD<br>Placebo	12 weeks	≥40 years of age; smoking history of ≥10 pack-years; diagnosis of COPD; moderate-to-severe airway obstruction with a pre-bronchodilator FEV_1 ≤60% predicted; FEV_1/FVC ≤70% (based on ECCS values)	Allowed: rescue salbutamol pressurized MDI as needed; oral corticosteroids (equivalent of <10 mg prednisone per day), orally inhaled corticosteroids, theophyllines and mucolytics if stabilized for at least 6 weeks prior to and throughout the study.

Study	Treatment	Trial duration	Inclusion criteria	Background treatment
				Not allowed: oral β-adrenergics and other investigational drugs (at least 1 month prior to run-in); cromolyn sodium and nedocromil sodium (at least 3 months prior to run-in); anticholinergics; inhaled β-adrenergics other than salbutamol or fixed combination inhalers

ACL, aclidinium; ATS, American Thoracic Society; BID, twice daily; COPD, chronic obstructive pulmonary disease; ECCS, European Community of Coal and Steel; FEV_1, forced expiratory volume in 1 second; FOR, formoterol; FP, fluticasone propionate; FVC, forced vital capacity; GLY, glycopyrronium; GOLD, Global Initiative for Chronic Obstructive Lung Disease; ICS, inhaled corticosteroid; IND, indacaterol; LABA, long-acting β_2-agonist; LAMA, long-acting muscarinic antagonist; MDI, metered dose inhaler; (m)MRC, (modified) Medical Research Council; NMA, network meta-analysis; NR, not reported; OD, once daily; OLO, olodaterol; SABA, short-acting β_2-agonist; SAL, salmeterol; SAMA, short-acting muscarinic antagonist; SSRI, selective serotonin reuptake inhibitor; SVC, slow vital capacity; TIO, tiotropium; UMEC, umeclidinium; VI, vilanterol.

Supplementary Table S4: Risk of bias assessment for included studies

Study	Adequate generation of randomization sequence	Adequate allocation concealment	Blinding		Result independent reporting of all relevant endpoints	Other aspects that increase the risk of bias	Overall rating of study specific risk of bias
			Patients	Caregivers			
DB2113373	Yes	Yes	Yes	Yes	Yes	No	Low
DB2113374	Yes	Yes	Yes	Yes	Yes	No	Low
DB2113360	Yes	Yes	Yes	Yes	Yes	No	Low
ZEP117115	Yes	Yes	Yes	Yes	Yes	No	Low
Aaron 2007	Yes	Yes	Yes	Yes	Yes	No	Low
Abrahams 2013 (NCT00528996)	Unclear	Unclear	Yes	Yes	Yes	No	Low
ACLIFORM COPD (Singh 2014)	Yes	Yes	Yes	Yes	Yes	No	Low
ANHELTO 1 (NCT01694771; ZuWallack 2014)	Unclear	Unclear	Yes	Yes	Yes	No	Low
ANHELTO 2 (NCT01696058; ZuWallack 2014)	Unclear	Unclear	Yes	Yes	Yes	No	Low
AUGMENT COPD (NCT01437397; D'Urzo 2014)	Unclear	Unclear	Yes	Yes	Yes	No	Low
Bateman 2010a (NCT00387088)	Yes	Yes	Yes	Yes	Yes	No	Low
Bateman 2010b (NCT00168844; NCT00168831)	Unclear	Unclear	Yes	Yes	Yes	No	Low
Beeh 2006	Unclear	Unclear	Yes	Yes	Yes	No	Low
BI trial 205.269 (NCT00144326)	Unclear	Unclear	Yes	Yes	Yes	No	Low
Brusasco 2003	Unclear	Unclear	Yes	Yes	Yes	No	Low

Study	Adequate generation of randomization sequence	Adequate allocation concealment	Blinding		Result independent reporting of all relevant endpoints	Other aspects that increase the risk of bias	Overall rating of study specific risk of bias
			Patients	Caregivers			
Casaburi 2000	Unclear	Unclear	Yes	Yes	Yes	No	Low
Casaburi 2002	Unclear	Unclear	Yes	Yes	Yes	No	Low
Chan 2007 (BI trial: 205.259)	Unclear	Unclear	Yes	Yes	Yes	No	Low
Covelli 2005	Unclear	Unclear	Yes	Yes	Yes	No	Low
Donohue 2002	Unclear	Unclear	Yes	Yes	Yes	No	Low
Donohue 2010	Unclear	Unclear	No	No	Yes	No	High
ENLIGHTEN (Dahl 2013)	Unclear	Unclear	Yes	Yes	Yes	Yes	Low
FLIGHT1 (NCT01727141; Mahler 2015)	Yes	Yes	Yes	Yes	Yes	No	Low
FLIGHT2 (NCT01712516; Mahler 2015)	Yes	Yes	Yes	Yes	Yes	No	Low
GLOW2 (Kerwin 2012)	Unclear	Unclear	Yes	Yes	Yes	No	Low
INTRUST-1 (Mahler 2012)	Yes	Yes	Yes	Yes	Yes	No	Low
INTRUST-2 (Mahler 2012)	Yes	Yes	Yes	Yes	Yes	No	Low
Moita 2008	Unclear	Unclear	Yes	Yes	Yes	No	Low
Niewoehner 2005	Yes	Yes	Yes	Yes	Yes	No	Low
OTEMTO 1 (Singh 2015)	Unclear	Unclear	Yes	Yes	Yes	No	Low
OTEMTO 2 (Singh 2015)	Unclear	Unclear	Yes	Yes	Yes	No	Low
QUANTIFY (NCT01120717; Buhl 2015)	Yes	Yes	Yes	Yes	Yes	No	Low
SHINE (Bateman 2013)	Yes	No	No	No	Yes	No	High
SPARK (Wedzicha 2013)	Yes	No	No	No	No	No	High
Tashkin 2009	Yes	Yes	Yes	Yes	Yes	No	Low

Study	Adequate generation of randomization sequence	Adequate allocation concealment	Blinding		Result independent reporting of all relevant endpoints	Other aspects that increase the risk of bias	Overall rating of study specific risk of bias
			Patients	Caregivers			
TIOSPIR (NCT01126437; Wise 2013; Anzueto 2015)	Yes	Yes	Yes	Yes	Yes	No	Low
TIPHON (Tonnel 2008)	Yes	Yes	Yes	Yes	Yes	No	Low
TONADO-1 (NCT01431274; Buhl 2015; Ferguson 2015)	Yes	Yes	Yes	Yes	Yes	No	Low
TONADO-2 (NCT01431287; Buhl 2015; Ferguson 2015)	Yes	Yes	Yes	Yes	Yes	No	Low
Troosters 2014 (NCT00523991)	Unclear	Unclear	Yes	Yes	Yes	No	Low
UPLIFT (Tashkin 2008, 2012 and 2014; Celli 2009; Buhl 2012)	Yes	Yes	Yes	Yes	Yes	No	Low
Verkindre 2006	Unclear	Unclear	Yes	Yes	Yes	No	Low
Vogelmeier 2008 (NCT00134979)	Unclear	No	No	No	Yes	No	High
Voshaar 2008 (NCT00239473, BI study number 205.251; NCT00240435, BI study number 205.252)	Unclear	Unclear	Yes	Yes	Yes	No	Low

Supplementary Table S5: Individual study results at 12 and 24 weeks for trough FEV_1, SGRQ total scores, TDI focal scores, and rescue medication use (puffs/day)

Study	Treatment	Weeks	Trough FEV_1 in mL (difference in CFB), mean (SE)	SGRQ total score (difference in CFB), mean (SE)	TDI focal score (difference in TDI), mean (SE)	Rescue medication use (difference in puffs/day), mean (SE)
DB2113373	UMEC/VI 62.5/25 vs PBO	12	195.00 (17.86)	-4.72 (1.06)	1.30 (0.23)	-1.00 (0.24)
		24	167.00 (20.15)	-5.51 (1.21)	1.20 (0.26)	-0.80 (0.26)
DB2113374	UMEC/VI 62.5/25 vs TIO 18	12	95.00 (21.94)	-2.01 (1.26)	0.70 (0.31)	-0.51 (0.32)
		24	60.00 (25.26)	-0.17 (1.37)	0.20 (0.36)	-0.60 (0.31)
DB2113360	UMEC/VI 62.5/25 vs TIO 18	12	80.00 (24.49)	-0.23 (1.29)	0.20 (0.26)	-0.78 (0.30)
		24	90.00 (26.02)	0.75 (1.47)	-0.10 (0.31)	-0.70 (0.28)
ZEP117115	UMEC/VI 62.5/25 vs TIO 18	12	109.00 (15.82)	-2.08 (0.70)		-0.50 (0.10)
		24	112.00 (16.07)	-2.10 (0.77)		-0.50 (0.13)
ACLIFORM	ACL/FOR 400/12 vs PBO	12	127.10 (17.38[a])			
		24	143.00 (12.00)	-0.65 (1.24)	1.29 (0.29)	-0.66 (0.20[a])
AUGMENT	ACL/FOR 400/12 vs PBO	12	146.00 (18.55[a])			
		24	129.00 (17.54)	-4.36 (1.05)	1.44 (0.27[a])	
OTEMTO 1	TIO/OLO 5/5 vs PBO	12	162.00 (19.00)	-4.89 (1.02)	2.05 (0.27)	
		24				
OTEMTO 2	TIO/OLO 5/5 vs PBO	12	166.00 (19.00)	-4.56 (0.99)	1.20 (0.27)	
		24				

Study	Treatment	Weeks	Trough FEV_1 in mL (difference in CFB), mean (SE)	SGRQ total score (difference in CFB), mean (SE)	TDI focal score (difference in TDI), mean (SE)	Rescue medication use (difference in puffs/day), mean (SE)
TONADO-1	TIO/OLO 5/5 vs TIO 5	12	75.60 (14.92[a])			-0.68 (0.11[a])[b]
		24	71.00 (12.00)	-1.23 (0.55)[b]	0.36 (0.14)[b]	-0.63 (0.12[a])[b]
TONADO-2	TIO/OLO 5/5 vs TIO 5	12	59.30 (15.14[a])			
		24	50.00 (13.00)			
ANHELTO 1	OLO 5 + TIO 18	12	62.00 (13.00)	-1.85 (0.46)[c]		
		24				
ANHELTO 2	OLO 5 + TIO 18	12	40.00 (13.00)			
		24				
INTRUST-1	IND 150 + TIO 18 vs TIO 18	12	80.00 (12.76)			-1.10 (0.18)
		24				
INTRUST-2	IND 150 + TIO 18 vs TIO 18	12	70.00 (10.20)			-0.70 (0.15)
		24				
Aaron 2007	TIO 18 + SAL 50 vs TIO 18	12				
		24	18.49 (45.46)	-1.47 (0.69)	-0.42 (0.43)	
FLIGHT-1	GLY/IND 27.5/15.6 vs PBO	12	213.00 (20.66)	-3.80 (0.99)	1.23 (0.25)	-1.22 (0.18)
		24				
FLIGHT-2	GLY/IND 27.5/15.6 vs PBO	12	233.00 (20.66)	-6.40 (1.10)	2.03 (0.26)	-1.16 (0.19)
		24				

Study	Treatment	Weeks	Trough FEV_1 in mL (difference in CFB), mean (SE)	SGRQ total score (difference in CFB), mean (SE)	TDI focal score (difference in TDI), mean (SE)	Rescue medication use (difference in puffs/day), mean (SE)
ENLIGHTEN	GLY/IND 110/50 vs PBO	12	163.00 (32.02)			
		24	152.00 (35.36)			
QUANTIFY	GLY/IND 110/50 vs TIO 18 + FOR 12	12	72.00 (16.33)			
		24	68.00 (16.07)		0.38 (0.22)	
SPARK	GLY/IND 110/50 vs TIO 18	12	70.00 (13.79)	-3.00 (0.88)		
		24	70.00 (13.79)	-1.60 (0.92)		
SHINE	GLY/IND 110/50 vs PBO	12	230.00 (17.86)	-3.99 (0.76[a])	1.22 (0.26)	
		24	200.00 (17.86)	-3.01 (1.04)	1.09 (0.24)	-0.96 (0.17)
SHINE	TIO 18 vs PBO	12	130.00 (17.86)	-2.37 (0.76[a])	0.59 (0.27)	
		24	130.00 (17.86)	-0.88 (1.04)	0.58 (0.24)	-0.41 (0.17)
Tashkin 2009	TIO 18 + FOR 12 vs TIO 18	12	90.00 (28.06)	-1.01 (1.49[a])	0.07 (0.39)	-0.25 (0.32[a])
		24				
Vogelmeier 2008	TIO 18 + FOR 10 vs PBO	12				
		24		-2.93 (1.33)		
Vogelmeier 2008	TIO 18 + FOR 10 vs TIO 18	12				
		24		-0.88 (1.84)		
Vogelmeier 2008	TIO 18 vs PBO	12				
		24		-2.05 (1.27)		

Study	Treatment	Weeks	Trough FEV_1 in mL (difference in CFB), mean (SE)	SGRQ total score (difference in CFB), mean (SE)	TDI focal score (difference in TDI), mean (SE)	Rescue medication use (difference in puffs/day), mean (SE)
Beeh 2006	TIO 18 vs PBO	12	79.00 (17.00)			
		24				
Brusasco 2003	TIO 18 vs PBO	12				
		24	120.00 (100.00)	-2.7 (0.99)	1.10 (0.30)	
Casaburi 2000	TIO 18 vs PBO	12	150.00 (14.00)			
		24				
Casaburi 2002	TIO 18 vs PBO	12	133.00 (14.54[a])		0.95 (0.18)	
		24	170.00 (18.65[a])	-3.08 (0.83[a])	0.85 (0.19)	
Chan 2007	TIO 18 vs PBO	12	100.00 (15.00)			
		24				
Covelli 2005	TIO 18 vs PBO	12	184.00 (37.00)			
		24				
Donohue 2002	TIO 18 vs PBO	12				
		24	137.00 (20.00)	-2.71 (1.34[a])	1.02 (0.35[a])	-1.45 (0.28[a])
Donohue 2010	TIO 18 vs PBO	12	140.00 (20.41)	-1.10 (0.87)	0.75 (0.22)	
		24	140.00 (20.41)	-1.00 (0.92)	0.87 (0.23)	-0.60 (0.19)
GLOW2	TIO 18 vs PBO	12	83.00 (19.00)	-2.84 (0.97)	0.26 (0.30)	
		24	84.00 (21.60)	-2.52 (1.11)	0.94 (0.30)	

Study	Treatment	Weeks	Trough FEV_1 in mL (difference in CFB), mean (SE)	SGRQ total score (difference in CFB), mean (SE)	TDI focal score (difference in TDI), mean (SE)	Rescue medication use (difference in puffs/day), mean (SE)
Moita 2008	TIO 18 vs PBO	12	102.00 (31.38)			
		24				
Niewoehner 2005	TIO 18 vs PBO	12	100.00 (10.00)			
		24	100.00 (13.00)			
TIPHON	TIO 18 vs PBO	12		-3.59 (1.22)		
		24		-3.51 (0.65)		
Troosters 2014	TIO 18 vs PBO	12				
		24	140.00 (22.96)			
UPLIFT	TIO 18 vs PBO	12				
		24	100.00 (7.00)	-2.5 (0.36)		
Verkindre 2006	TIO 18 vs PBO	12	110.00 (40.00)	-6.50 (2.90)	1.28 (0.89)	-0.13 (0.25)
		24				
Abrahams 2012/2013	TIO 5 vs PBO	12	119.95 (14.97)	-3.30 (0.28)	0.68 (0.18)	
		24	126.10 (15.14)	-3.50 (0.94[a])	0.52 (0.23)	
Bateman 2010a	TIO 5 vs PBO	12				
		24	103.00 (7.65)	-2.20 (0.46[a])		
Bateman 2010b	TIO 5 vs PBO	12				
		24	123.10 (16.89[a])			

Study	Treatment	Weeks	Trough FEV_1 in mL (difference in CFB), mean (SE)	SGRQ total score (difference in CFB), mean (SE)	TDI focal score (difference in TDI), mean (SE)	Rescue medication use (difference in puffs/day), mean (SE)
OTEMTO 1	TIO 5 vs PBO	12	134.00 (19.00)	-2.40 (1.03)	1.45 (0.27)	
		24				
OTEMTO 2	TIO 5 vs PBO	12	127.00 (19.00)	-2.85 (0.99)	0.61 (0.27)	
		24				
Voshaar 2008	TIO 5 vs PBO	12	118.00 (23.00)			
		24				

[a]Imputed value; [b]pooled data for TONADO-1 and TONADO-2 studies; [c]pooled data for ANHELTO 1 and ANHELTO 2 studies.
ACL, aclidinium; CFB, change from baseline; FEV_1, forced expiratory volume in 1 second; FOR, formoterol; GLY, glycopyrronium; IND, indacaterol; OLO, olodaterol; PBO, placebo; SAL, salmeterol; SE, standard error; SGRQ, St George's Respiratory Questionnaire; TDI, Transition Dyspnea Index; TIO, tiotropium; UMEC, umeclidinium; VI, vilanterol.

Supplementary Table S6: Results of the base case network meta-analysis for trough FEV_1 at 12 weeks (difference in change from baseline, mL)[a]

Intervention		Comparator								
		Placebo	TIO 5 or 18	TIO 18 + FOR 12	TIO 18 + IND 150	ACL/FOR 400/12	TIO 18 + OLO 5	TIO/OLO 5/5	GLY/IND 27.5/15.6	GLY/IND 110/50
TIO 5 or 18	Estimate	114.70								
	95% CrI	106.20 123.40								
	P(better)	>99%								
TIO 18 + FOR 12	Estimate	151.60	36.89							
	95% CrI	118.80 183.10	5.01 67.67							
	P(better)	>99%	99%							
TIO 18 + IND 150	Estimate	188.60	73.77	37.09						
	95% CrI	170.20 207.30	57.48 90.08	2.37 72.66						
	P(better)	>99%	>99%	98%						
ACL/FOR 400/12	Estimate	135.90	21.01	-15.61	-52.72					
	95% CrI	110.60 160.80	-5.06 47.44	-55.85 25.73	-83.79 -21.59					
	P(better)	>99%	94%	22%	0%					
TIO 18 + OLO 5	Estimate	165.70	50.90	14.31	-22.89	30.06				
	95% CrI	145.30 186.20	32.23 69.26	-21.77 50.65	-47.25 2.52	-2.58 61.66				
	P(better)	>99%	>99%	78%	4%	96%				
TIO/OLO 5/5	Estimate	170.20	55.48	18.77	-18.29	34.48	4.54			
	95% CrI	153.50 186.90	40.03 71.33	-15.53 53.81	-41.10 4.55	4.27 63.85	-19.76 29.22			
	P(better)	>99%	>99%	85%	6%	99%	64%			
GLY/IND 27.5/15.6	Estimate	222.20	107.30	70.51	33.46	86.29	56.55	51.80		
	95% CrI	193.30 250.90	77.40 137.50	28.03 113.30	-0.43 67.90	48.84 124.80	21.38 91.97	18.21 85.51		
	P(better)	>99%	>99%	>99%	97%	>99%	>99%	>99%		
GLY/IND 110/50	Estimate	205.10	90.27	53.57	16.56	69.37	39.16	34.79	-17.06	
	95% CrI	185.70 224.00	72.03 108.60	25.22 82.05	-8.05 40.60	37.30 100.50	13.24 65.23	11.14 58.55	-51.55 17.17	
	P(better)	>99%	>99%	>99%	91%	>99%	>99%	>99%	17%	
UMEC/VI 62.5/25	Estimate	208.10	93.23	56.11	19.43	72.05	42.21	37.63	-14.26	2.93
	95% CrI	187.90 228.20	73.60 112.50	20.83 93.78	-6.08 44.53	39.81 104.20	15.90 69.38	12.88 62.71	-49.18 21.33	-22.66 29.09
	P(better)	>99%	>99%	>99%	93%	>99%	>99%	>99%	22%	58%

[a]Where the probability of improved outcomes using therapy A vs therapy B is X%, the probability of improved outcomes using therapy B vs therapy A is (100-X)%.

ACL, aclidinium; CrI, credible interval; FEV_1, forced expiratory volume in 1 second; FOR, formoterol; GLY, glycopyrronium; IND, indacaterol; OLO, olodaterol; TIO, tiotropium; UMEC, umeclidinium; VI, vilanterol.

Supplementary Table S7: Results of the base case network meta-analysis for trough FEV_1 at 24 weeks (difference in change from baseline, mL)[a]

Intervention		Comparator						
		Placebo	TIO 5 or 18	TIO 18 + SAL 50	TIO 18 + FOR 12	ACL/FOR 400/12	TIO/OLO 5/5	GLY/IND 110/50
TIO 5 or 18	Estimate	110.90						
	95% CrI	102.90 118.90						
	P(better)	>99%						
TIO 18 + SAL 50	Estimate	123.60	12.72					
	95% CrI	39.40 215.90	-71.21 104.50					
	P(better)	>99%	61%					
TIO 18 + FOR 12	Estimate	113.50	2.64	-10.20				
	95% CrI	76.33 150.40	-34.16 39.06	-109.10 81.73				
	P(better)	>99%	56%	42%				
ACL/FOR 400/12	Estimate	138.20	27.33	15.03	24.64			
	95% CrI	117.90 158.30	5.65 48.95	-80.30 101.60	-17.54 67.69			
	P(better)	>99%	>99%	62%	87%			
TIO/OLO 5/5	Estimate	172.10	61.21	48.59	58.54	33.83		
	95% CrI	152.70 191.70	43.54 79.01	-45.86 133.50	18.22 99.60	5.96 62.05		
	P(better)	>99%	>99%	85%	>99%	98%		
GLY/IND 110/50	Estimate	181.80	70.99	58.24	68.16	43.63	9.66	
	95% CrI	162.50 200.90	52.76 89.04	-35.31 143.60	36.93 100.10	15.72 71.35	-15.53 35.11	
	P(better)	>99%	>99%	89%	>99%	>99%	78%	
UMEC/VI 62.5/25	Estimate	195.80	84.92	72.27	82.43	57.47	23.74	13.87
	95% CrI	174.80 217.30	64.61 105.50	-21.21 158.20	40.01 124.10	28.76 87.14	-3.31 50.73	-12.98 41.33
	P(better)	>99%	>99%	94%	>99%	>99%	96%	84%

[a]Where the probability of improved outcomes using therapy A vs therapy B is X%, the probability of improved outcomes using therapy B vs therapy A is (100-X)%.

ACL, aclidinium; CrI, credible interval; FEV_1, forced expiratory volume in 1 second; FOR, formoterol; GLY, glycopyrronium; IND, indacaterol; OLO, olodaterol; SAL, salmeterol; TIO, tiotropium; UMEC, umeclidinium; VI, vilanterol.

Supplementary Table S8: Results of the base case network meta-analysis for SGRQ total score at 12 weeks (difference in change from baseline)[a]

Intervention		Comparator						
		Placebo	TIO 5 or 18	TIO 18 + FOR 12	TIO 18 + OLO 5	TIO/OLO 5/5	GLY/IND 27.5/15.6	GLY/IND 110/50
TIO 5 or 18	Estimate	-2.97						
	95% CrI	-3.41 -2.53						
	P(better)	>99%						
TIO 18 + FOR 12	Estimate	-3.99	-1.01					
	95% CrI	-6.94 -1.03	-3.94 1.89					
	P(better)	>99%	75%					
TIO 18 + OLO 5	Estimate	-4.82	-1.85	-0.84				
	95% CrI	-5.82 -3.82	-2.75 -0.95	-3.90 2.24				
	P(better)	>99%	>99%	71%				
TIO/OLO 5/5	Estimate	-4.90	-1.93	-0.90	-0.07			
	95% CrI	-6.10 -3.68	-3.13 -0.71	-4.07 2.26	-1.58 1.43			
	P(better)	>99%	>99%	71%	54%			
GLY/IND 27.5/15.6	Estimate	-4.97	-2.01	-0.99	-0.15	-0.08		
	95% CrI	-6.42 -3.52	-3.51 -0.49	-4.27 2.30	-1.91 1.61	-1.96 1.82		
	P(better)	>99%	>99%	72%	57%	53%		
GLY/IND 110/50	Estimate	-4.89	-1.92	-0.91	-0.07	0.00	0.08	
	95% CrI	-5.98 -3.80	-2.97 -0.87	-3.99 2.21	-1.46 1.32	-1.59 1.59	-1.73 1.90	
	P(better)	>99%	>99%	72%	54%	50%	47%	
UMEC/VI 62.5/25	Estimate	-4.70	-1.73	-0.72	0.12	0.20	0.27	0.19
	95% CrI	-5.72 -3.68	-2.70 -0.77	-3.79 2.36	-1.20 1.44	-1.35 1.71	-1.50 2.04	-1.23 1.61
	P(better)	>99%	>99%	68%	43%	40%	38%	40%

[a]Where the probability of improved outcomes using therapy A vs therapy B is X%, the probability of improved outcomes using therapy B vs therapy A is (100-X)%.

CrI, credible interval; FOR, formoterol; GLY, glycopyrronium; IND, indacaterol; OLO, olodaterol; SGRQ, St George's Respiratory Questionnaire; TIO, tiotropium; UMEC, umeclidinium; VI, vilanterol.

Supplementary Table S9: Results of the base case network meta-analysis for SGRQ total score at 24 weeks (difference in change from baseline)[a]

Intervention		Comparator						
		Placebo	TIO 5 or 18	TIO 18 + FOR 10	TIO 18 + SAL 50	ACL/FOR 400/12	TIO/OLO 5/5	GLY/IND 110/50
TIO 5 or 18	Estimate	-2.53						
	95% CrI	-2.94 -2.12						
	P(better)	>99%						
TIO 18 + FOR 10	Estimate	-3.18	-0.64					
	95% CrI	-5.46 -0.89	-2.93 1.64					
	P(better)	>99%	71%					
TIO 18 + SAL 50	Estimate	-4.00	-1.47	-0.83				
	95% CrI	-5.42 -2.59	-2.83 -0.11	-3.49 1.84				
	P(better)	>99%	98%	73%				
ACL/FOR 400/12	Estimate	-2.80	-0.27	0.37	1.20			
	95% CrI	-4.37 -1.22	-1.89 1.36	-2.40 3.15	-0.92 3.32			
	P(better)	>99%	63%	40%	13%			
TIO/OLO 5/5	Estimate	-3.76	-1.23	-0.58	0.24	-0.96		
	95% CrI	-4.92 -2.60	-2.32 -0.14	-3.11 1.95	-1.49 1.97	-2.91 0.99		
	P(better)	>99%	99%	68%	39%	83%		
GLY/IND 110/50	Estimate	-4.29	-1.76	-1.12	-0.28	-1.49	-0.53	
	95% CrI	-5.34 -3.23	-2.75 -0.76	-3.59 1.39	-1.97 1.40	-3.38 0.40	-2.00 0.95	
	P(better)	>99%	>99%	81%	63%	94%	76%	
UMEC/VI 62.5/25	Estimate	-4.11	-1.58	-0.93	-0.10	-1.31	-0.35	0.18
	95% CrI	-5.23 -2.99	-2.65 -0.51	-3.45 1.59	-1.84 1.63	-3.24 0.60	-1.87 1.17	-1.28 1.63
	P(better)	>99%	>99%	77%	55%	91%	67%	40%

[a]Where the probability of improved outcomes using therapy A vs therapy B is X%, the probability of improved outcomes using therapy B vs therapy A is (100-X)%.

ACL, aclidinium; CrI, credible interval; FOR, formoterol; GLY, glycopyrronium; IND, indacaterol; OLO, olodaterol; SAL, salmeterol; SGRQ, St George's Respiratory Questionnaire; TIO, tiotropium; UMEC, umeclidinium; VI, vilanterol.

Supplementary Table S10: Results of the base case network meta-analysis for TDI focal score at 12 weeks (difference in TDI)[a]

Intervention		Comparator					
		Placebo	TIO 5 or 18	TIO 18 + FOR 12	TIO/OLO 5/5	GLY/IND 27.5/15.6	GLY/IND 110/50
TIO 5 or 18	Estimate	0.79					
	95% CrI	0.52 1.06					
	P(better)	>99%					
TIO 18 + FOR 12	Estimate	0.86	0.07				
	95% CrI	-0.12 1.85	-0.88 1.02				
	P(better)	96%	56%				
TIO/OLO 5/5	Estimate	1.50	0.71	0.64			
	95% CrI	1.01 2.00	0.22 1.21	-0.43 1.71			
	P(better)	>99%	>99%	89%			
GLY/IND 27.5/15.6	Estimate	1.62	0.83	0.76	0.12		
	95% CrI	1.07 2.17	0.22 1.45	-0.36 1.89	-0.62 0.86		
	P(better)	>99%	>99%	91%	64%		
GLY/IND 110/50	Estimate	1.34	0.55	0.48	-0.16	-0.28	
	95% CrI	0.67 1.99	-0.11 1.20	-0.68 1.63	-0.97 0.63	-1.14 0.57	
	P(better)	>99%	96%	80%	33%	24%	
UMEC/VI 62.5/25	Estimate	1.24	0.45	0.38	-0.25	-0.37	-0.10
	95% CrI	0.77 1.73	0.00 0.92	-0.68 1.44	-0.91 0.40	-1.11 0.36	-0.87 0.70
	P(better)	>99%	97%	77%	20%	14%	39%

[a]Where the probability of improved outcomes using therapy A vs therapy B is X%, the probability of improved outcomes using therapy B vs therapy A is (100-X)%.

CrI, credible interval; FOR, formoterol; GLY, glycopyrronium; IND, indacaterol; OLO, olodaterol; TDI, Transition Dyspnea Index; TIO, tiotropium; UMEC, umeclidinium; VI, vilanterol.

Supplementary Table S11: Results of the base case network meta-analysis for TDI focal score at 24 weeks (difference in TDI)[a]

Intervention		Comparator						
		Placebo	TIO 5 or 18	TIO 18 + SAL 50	TIO 18 + FOR 12	ACL/FOR 400/12	TIO/OLO 5/5	GLY/IND 110/50
TIO 5 or 18	Estimate	0.83						
	95% CrI	0.65 1.01						
	P(better)	>99%						
TIO 18 + SAL 50	Estimate	0.41	-0.42					
	95% CrI	-0.46 1.27	-1.27 0.42					
	P(better)	82%	16%					
TIO 18 + FOR 12	Estimate	0.93	0.10	0.52				
	95% CrI	0.41 1.44	-0.40 0.59	-0.46 1.50				
	P(better)	>99%	65%	85%				
ACL/FOR 400/12	Estimate	1.37	0.54	0.96	0.44			
	95% CrI	0.98 1.76	0.12 0.97	0.02 1.91	-0.21 1.09			
	P(better)	>99%	>99%	98%	91%			
TIO/OLO 5/5	Estimate	1.19	0.36	0.78	0.26	-0.18		
	95% CrI	0.87 1.51	0.09 0.63	-0.10 1.67	-0.30 0.82	-0.68 0.32		
	P(better)	>99%	>99%	96%	82%	24%		
GLY/IND 110/50	Estimate	1.31	0.48	0.90	0.38	-0.06	0.12	
	95% CrI	1.03 1.58	0.25 0.71	0.03 1.78	-0.06 0.82	-0.54 0.41	-0.23 0.47	
	P(better)	>99%	>99%	98%	96%	40%	75%	
UMEC/VI 62.5/25	Estimate	1.01	0.18	0.61	0.08	-0.36	-0.18	-0.30
	95% CrI	0.66 1.36	-0.16 0.53	-0.31 1.52	-0.52 0.68	-0.88 0.16	-0.61 0.26	-0.71 0.11
	P(better)	>99%	85%	90%	61%	9%	21%	8%

[a]Where the probability of improved outcomes using therapy A vs therapy B is X%, the probability of improved outcomes using therapy B vs therapy A is (100-X)%.

ACL, aclidinium; CrI, credible interval; FOR, formoterol; GLY, glycopyrronium; IND, indacaterol; OLO, olodaterol; SAL, salmeterol; TDI, Transition Dyspnea Index; TIO, tiotropium; UMEC, umeclidinium; VI, vilanterol.

Supplementary Table S12: Results of the base case network meta-analysis for rescue medication use at 12 weeks (difference in change from baseline, puffs/day)[a]

Intervention		Comparator					
		Placebo	TIO 5 or 18	TIO 18 + FOR 12	TIO 18 + IND 150	TIO/OLO 5/5	GLY/IND 27.5/15.6
TIO 5 or 18	Estimate	-0.29					
	95% CrI	-0.64 0.06					
	P(better)	95%					
TIO 18 + FOR 12	Estimate	-0.54	-0.25				
	95% CrI	-1.26 0.17	-0.87 0.37				
	P(better)	93%	78%				
TIO 18 + IND 150	Estimate	-1.16	-0.87	-0.62			
	95% CrI	-1.58 -0.74	-1.10 -0.64	-1.28 0.04			
	P(better)	>99%	>99%	97%			
TIO/OLO 5/5	Estimate	-0.97	-0.68	-0.43	0.19		
	95% CrI	-1.38 -0.56	-0.89 -0.46	-1.09 0.22	-0.12 0.50		
	P(better)	>99%	>99%	90%	12%		
GLY/IND 27.5/15.6	Estimate	-1.19	-0.90	-0.65	-0.03	-0.22	
	95% CrI	-1.45 -0.93	-1.33 -0.46	-1.41 0.11	-0.52 0.46	-0.70 0.26	
	P(better)	>99%	>99%	95%	55%	81%	
UMEC/VI 62.5/25	Estimate	-0.84	-0.55	-0.30	0.32	0.13	0.35
	95% CrI	-1.19 -0.49	-0.73 -0.38	-0.94 0.34	0.03 0.61	-0.15 0.41	-0.09 0.78
	P(better)	>99%	>99%	82%	2%	18%	6%

[a]Where the probability of improved outcomes using therapy A vs therapy B is X%, the probability of improved outcomes using therapy B vs therapy A is (100-X)%.

CrI, credible interval; FOR, formoterol; GLY, glycopyrronium; IND, indacaterol; OLO, olodaterol; TIO, tiotropium; UMEC, umeclidinium; VI, vilanterol.

Supplementary Table S13: Results of the base case network meta-analysis for rescue medication use at 24 weeks (difference in change from baseline, puffs/day)[a]

Intervention		Comparator									
		Placebo		TIO 5 or 18		ACL/FOR 400/12		TIO/OLO 5/5		GLY/IND 110/50	
TIO 5 or 18	Estimate	-0.65									
	95% CrI	-1.26	-0.11								
	P(better)	99%									
ACL/FOR 400/12	Estimate	-0.66		-0.01							
	95% CrI	-1.75	0.44	-1.22	1.26						
	P(better)	92%		51%							
TIO/OLO 5/5	Estimate	-1.28		-0.63		-0.62					
	95% CrI	-2.52	-0.12	-1.69	0.42	-2.28	0.96				
	P(better)	98%		92%		85%					
GLY/IND 110/50	Estimate	-1.09		-0.43		-0.42		0.19			
	95% CrI	-2.07	-0.12	-1.39	0.56	-1.90	1.03	-1.22	1.67		
	P(better)	98%		87%		78%		34%			
UMEC/VI 62.5/25	Estimate	-1.13		-0.48		-0.47		0.15		-0.05	
	95% CrI	-1.86	-0.46	-1.05	0.10	-1.80	0.80	-1.04	1.37	-1.16	1.03
	P(better)	>99%		96%		83%		35%		55%	

[a]Where the probability of improved outcomes using therapy A vs therapy B is X%, the probability of improved outcomes using therapy B vs therapy A is (100-X)%.

ACL, aclidinium; CrI, credible interval; FOR, formoterol; GLY, glycopyrronium; IND, indacaterol; OLO, olodaterol; TIO, tiotropium; UMEC, umeclidinium; VI, vilanterol.

Supplementary Table S14: Results of the network meta-analysis for trough FEV_1, at 24 weeks (difference in CFB, mL [95% CrI]) for ICS users and ICS non-users[a]

Intervention		Comparator									
		Placebo		TIO 5 or 18		TIO 18 + FOR 12		TIO/OLO 5/5		GLY/IND 110/50	
		ICS users	ICS non-users	ICS users	ICS non-users	ICS users	ICS non-users	ICS users	ICS non-users	ICS users	ICS non-users
TIO 5 or 18	Estimate	109.40	159.00								
	95% CrI	(68.88, 150.60)	(111.40, 207.10)								
	P(better)	>99%	>99%								
TIO 18 + FOR 12	Estimate	118.30	161.50	8.69	2.56						
	95% CrI	(50.07, 186.70)	(93.60, 229.80)	(-53.09, 71.19)	(-56.94, 62.20)						
	P(better)	>99%	>99%	61%	53%						
TIO/OLO 5/5	Estimate	154.40	234.90	44.99	75.96	36.21	73.35				
	95% CrI	(107.70, 201.90)	(182.10, 288.60)	(21.53, 68.56)	(52.50, 99.53)	(-30.45, 102.60)	(9.13, 137.20)				
	P(better)	>99%	>99%	>99%	>99%	86%	99%				
GLY/IND 110/50	Estimate	178.80	238.10	69.40	79.22	60.54	76.58	24.42	3.29		
	95% CrI	(134.20, 223.80)	(185.70, 290.80)	(34.16, 104.50)	(38.16, 120.00)	(8.85, 111.90)	(32.89, 120.00)	(-18.36, 66.68)	(-44.38, 50.40)		
	P(better)	>99%	>99%	>99%	>99%	99%	>99%	87%	55%		
UMEC/VI 62.5/25	Estimate	179.50	279.80	69.99	120.90	61.16	118.20	24.95	44.87	0.67	41.72
	95% CrI	(127.00, 232.30)	(221.40, 338.40)	(36.72, 103.30)	(87.10, 154.70)	(-9.49, 131.70)	(49.53, 186.90)	(-16.04, 65.67)	(3.46, 86.00)	(-47.73, 48.95)	(-11.33, 94.79)
	P(better)	>99%	>99%	>99%	>99%	95%	>99%	88%	98%	51%	94%

[a]Where the probability of improved outcomes using therapy A vs therapy B is X%, the probability of improved outcomes using therapy B vs therapy A is (100-X)%.

CFB, change from baseline; CrI, credible interval; FEV_1, forced expiratory volume in 1 second; FOR, formoterol; GLY, glycopyrronium; ICS, inhaled corticosteroid; IND, indacaterol; OLO, olodaterol; TIO, tiotropium; UMEC, umeclidinium; VI, vilanterol.

Supplementary Table S15: Results of the network meta-analysis for trough FEV_1, at 24 weeks (difference in CFB, mL [95% CrI]) for patients with moderate and severe disease[a]

Intervention		Comparator									
		Placebo		TIO 5 or 18		TIO 18 + FOR 12		TIO/OLO 5/5		GLY/IND 110/50	
		Moderate	Severe	Moderate	Severe	Moderate	Severe	Moderate	Severe	Moderate	Severe
TIO 5 or 18	Estimate 95% CrI	157.30 (128.40, 186.30)	39.16 (-12.86, 91.72)								
	P(better)	>99%	93%								
TIO 18 + FOR 12	Estimate 95% CrI	146.80 (88.52, 205.20)	58.46 (-16.95, 133.20)	-10.57 (-65.75, 45.15)	19.27 (-36.32, 75.20)						
	P(better)	>99%	94%	36%	75%						
TIO/OLO 5/5	Estimate 95% CrI	217.30 (177.60, 257.40)	100.10 (44.16, 156.90)	59.97 (32.55, 87.52)	60.98 (40.05, 82.00)	70.50 (8.60, 132.00)	41.78 (-18.08, 101.40)				
	P(better)	>99%	>99%	>99%	>99%	99%	91%				
GLY/IND 110/50	Estimate 95% CrI	230.30 (193.00, 267.70)	118.90 (65.22, 173.10)	73.08 (40.55, 105.50)	79.82 (59.17, 100.40)	83.52 (38.33, 128.40)	60.53 (8.41, 112.30)	13.08 (-29.81, 55.54)	18.83 (-10.74, 48.30)		
	P(better)	>99%	>99%	>99%	>99%	>99%	99%	73%	89%		
UMEC/VI 62.5/25	Estimate 95% CrI	276.10 (228.50, 324.10)	117.10 (57.39, 177.50)	118.80 (80.60, 157.10)	78.00 (48.20, 107.90)	129.30 (61.63, 196.70)	58.69 (-4.79, 121.80)	58.79 (11.45, 105.80)	16.98 (-19.68, 53.38)	45.79 (-4.40, 95.93)	-1.80 (-38.11, 34.44)
	P(better)	>99%	>99%	>99%	>99%	>99%	97%	>99%	82%	96%	46%

[a]Where the probability of improved outcomes using therapy A vs therapy B is X%, the probability of improved outcomes using therapy B vs therapy A is (100-X)%.
CFB, change from baseline; CrI, credible interval; FEV_1, forced expiratory volume in 1 second; FOR, formoterol; GLY, glycopyrronium; IND, indacaterol; OLO, olodaterol; TIO, tiotropium; UMEC, umeclidinium; VI, vilanterol.

References

1. Donohue JF, Maleki-Yazdi MR, Kilbride S, Mehta R, Kalberg C, Church A. Efficacy and safety of once-daily umeclidinium/vilanterol 62.5/25 mcg in COPD. Respir Med. 2013;107:1538–46.
2. GlaxoSmithKline. A 24-week Evaluation of GSK573719/Vilanterol (62.5/25mcg) and Components in COPD (NCT01313650). 2014. [Available from: http://ClinicalTrials.gov/show/NCT01313650]. Accessed 2 Oct 2015.
3. GlaxoSmithKline. A 24-Week, Randomized, Double-Blind, Placebo-Controlled Study to Evaluate the Efficacy and Safety of GSK573719/GW642444 Inhalation Powder and the Individual Components Delivered Once-Daily via a Novel Dry Powder Inhaler in Subjects with Chronic Obstructive Pulmonary Disease. 2014. [Available from: https://www.clinicaltrialsregister.eu/ctr-search/search?query=eudract_number:2010-023349-32]. Accessed 8 Oct 2015.
4. GlaxoSmithKline. 24-week Trial Comparing GSK573719/GW642444 With GSK573719 and With Tiotropium in Chronic Obstructive Pulmonary Disease. 2014. [Available from: http://ClinicalTrials.gov/show/NCT01316913]. Accessed 2 Oct 2015.
5. GlaxoSmithKline. A multicenter trial comparing the efficacy and safety of GSK573719/GW642444 with GSK573719 with tiotropium over 24 weeks in subjects with COPD (2010-021802-39). 2014. [Available from: https://www.clinicaltrialsregister.eu/ctr-search/search?query=2010-021802-39]. Accessed 8 Oct 2015.
6. GlaxoSmithKline. A Multicenter Trial Comparing the Efficacy and Safety of GSK573719/GW642444 with GSK573719 and withTiotropium over 24 Weeks in Subjects with COPD (DB2113374): Clinical Study Report. 2012.
7. GlaxoSmithKline. 24-week Trial Comparing GSK573719/GW642444 With GW642444 and With Tiotropium in Chronic Obstructive Pulmonary Disease. 2013. [Available from: http://ClinicalTrials.gov/show/NCT01316900]. Accessed 2 Oct 2015.
8. GlaxoSmithKline. DB2113360: A multicenter trial comparing the efficacy and safety of GSK573719/GW642444 with GW642444 and with tiotropium over 24 weeks in subjects with COPD. 2010-021800-72. 2014. [Available from: https://www.clinicaltrialsregister.eu/ctr-search/search?query=eudract_number:2010-021800-72]. Accessed 8 Oct 2015.
9. GlaxoSmithKline. A Multicenter Trial Comparing the Efficacy and Safety of GSK573719/GW642444 with GW642444 and with Tiotropium over 24 Weeks in Subjects with COPD (DB2113360): Clinical Study Report. 2012.
10. GlaxoSmithKline. The Purpose of This Study is to Evaluate the Spirometric Effect (Trough FEV1) of Umeclidinium/Vilanterol 62.5/25 mcg Once Daily Compared With Tiotriopium 18 mcg Once Daily Over a 24-week Treatment Period in Subjects With COPD (NCT01777334). 2013. [Available from: http://ClinicalTrials.gov/show/NCT01777334]. Accessed 2 Oct 2015.
11. GlaxoSmithKline. A Multicenter Trial Comparing the Efficacy and Safety of Umeclidinium/Vilanterol 62.5/25 mcg Once Daily with Tiotropium 18 mcg Once Daily over 24 Weeks in Subjects with Chronic Obstructive Pulmonary Disease (COPD). 2014. [Available from: https://www.clinicaltrialsregister.eu/ctr-search/search?query=eudract_number:2012-003973-24]. Accessed 8 Oct 2015.
12. GlaxoSmithKline. A Multicenter Trial Comparing the Efficacy and Safety of Umeclidinium/Vilanterol 62.5/25 mcg Once Daily with Tiotropium 18 mcg Once Daily over 24 Weeks in Subjects with Chronic Obstructive Pulmonary Disease (COPD) (ZEP117115): Clinical Study Report. 2013.
13. Aaron SD, Vandemheen KL, Fergusson D, et al. Tiotropium in combination with placebo, salmeterol, or fluticasone-salmeterol for treatment of chronic obstructive pulmonary disease: a randomized trial. Ann Intern Med. 2007;146:545–55.
14. OHRI. Optimal therapy of chronic obstructive pulmonary disease to prevent exacerbations and improve quality of life: a randomised, double-blind, placebo-controlled trial (ISRCTN29870041). 2006. [Available from: http://www.controlled-

trials.com/mrct/trial/1990427]. Accessed 8 Oct 2015. [Currently available from: http://www.isrctn.com/ISRCTN29870041]. Accessed 5 Jul 2017.
15. Abrahams R, Moroni-Zentgraf P, Ramsdell J, Schmidt H, Joseph E, Karpel J. Safety and efficacy of the once-daily anticholinergic BEA2180 compared with tiotropium in patients with COPD. Respir Med. 2013;107:854–62.
16. Singh D, Jones PW, Bateman ED, et al. Efficacy and safety of aclidinium bromide/formoterol fumarate fixed-dose combinations compared with individual components and placebo in patients with COPD (ACLIFORM-COPD): a multicentre, randomised study. BMC Pulm Med. 2014;14:178.
17. ZuWallack R, Allen L, Hernandez G, Ting N, Abrahams R. Efficacy and safety of combining olodaterol Respimat® and tiotropium HandiHaler® in patients with COPD: results of two randomized, double-blind, active-controlled studies. Int J Chron Obstruct Pulmon Dis. 2014;9:1133–44.
18. D'Urzo AD, Rennard SI, Kerwin EM, et al. Efficacy and safety of fixed-dose combinations of aclidinium bromide/formoterol fumarate: the 24-week, randomized, placebo-controlled AUGMENT COPD study. Respir Res. 2014;15:123.
19. Bateman ED, Tashkin D, Siafakas N, et al. A one-year trial of tiotropium Respimat plus usual therapy in COPD patients. Respir Med. 2010;104:1460–72.
20. Bateman E, Singh D, Smith D, et al. Efficacy and safety of tiotropium Respimat SMI in COPD in two 1-year randomized studies. Int J Chron Obstruct Pulmon Dis. 2010;5:197–208.
21. Beeh KM, Beier J, Buhl R, et al. [Efficacy of tiotropium bromide (Spiriva) in patients with chronic-obstructive pulmonary disease (COPD) of different severities]. Pneumologie. 2006;60:341–6.
22. Boehringer Ingelheim. A randomised, double-blind, placebo-controlled, 12 weeks trial to evaluate the effect of Tiotropium Inhalation Capsules on the magnitude of exercise, measured using an accelerometer, in patients with Chronic Obstructive Pulmonary Disease (COPD): Clinical Study Report Boehringer Ingelheim clinical trial registry. 2007. [Available from: http://www.trials.boehringer-ingelheim.com/public/trial_results_documents/205/205.269_CO.pdf]. Accessed 2 Oct 2015.
23. Brusasco V, Hodder R, Miravitlles M, Korducki L, Towse L, Kesten S. Health outcomes following treatment for six months with once daily tiotropium compared with twice daily salmeterol in patients with COPD. Thorax. 2003;58:399–404.
24. Casaburi R, Briggs DD, Jr., Donohue JF, Serby CW, Menjoge SS, Witek TJ, Jr. The spirometric efficacy of once-daily dosing with tiotropium in stable COPD: a 13-week multicenter trial. The US Tiotropium Study Group. Chest. 2000;118:1294–302.
25. Casaburi R, Mahler DA, Jones PW, et al. A long-term evaluation of once-daily inhaled tiotropium in chronic obstructive pulmonary disease. Eur Respir J. 2002;19:217–24.
26. Chan CK, Maltais F, Sigouin C, Haddon JM, Ford GT. A randomized controlled trial to assess the efficacy of tiotropium in Canadian patients with chronic obstructive pulmonary disease. Can Respir J. 2007;14:465–72.
27. Covelli H, Bhattacharya S, Cassino C, Conoscenti C, Kesten S. Absence of electrocardiographic findings and improved function with once-daily tiotropium in patients with chronic obstructive pulmonary disease. Pharmacotherapy. 2005;25:1708–18.
28. Donohue JF, van Noord JA, Bateman ED, et al. A 6-month, placebo-controlled study comparing lung function and health status changes in COPD patients treated with tiotropium or salmeterol. Chest. 2002;122:47–55.
29. Donohue JF, Fogarty C, Lotvall J, et al. Once-daily bronchodilators for chronic obstructive pulmonary disease: indacaterol versus tiotropium. Am J Respir Crit Care Med. 2010;182:155–62.
30. Dahl R, Chapman KR, Rudolf M, et al. Safety and efficacy of dual bronchodilation with QVA149 in COPD patients: the ENLIGHTEN study. Respir Med. 2013;107:1558–67.

31. Novartis. A Study to Assess the Long-term Safety of QVA149 (NCT01120717). 2013. [Available from: http://ClinicalTrials.gov/show/NCT01120717]. Accessed 2 Oct 2015.
32. Novartis. A multicenter, randomized, double-blind, placebo-controlled study, to assess the long term safety of 52 weeks treatment with QVA149 (110µg indacaterol / 50µg glycopyrrolate) in patients with moderate to severe Chronic Obstructive Pulmonary Disease (COPD). 2014. [Available from: https://www.clinicaltrialsregister.eu/ctr-search/search?query=eudract_number:2009-013235-38]. Accessed 8 Oct 2015.
33. Mahler DA, Kerwin E, Ayers T, et al. FLIGHT1 and FLIGHT2: Efficacy and Safety of QVA149 (Indacaterol/Glycopyrrolate) versus Its Monocomponents and Placebo in Patients with Chronic Obstructive Pulmonary Disease. Am J Respir Crit Care Med. 2015;192:1068–79.
34. Kerwin E, Hebert J, Gallagher N, et al. Efficacy and safety of NVA237 versus placebo and tiotropium in patients with COPD: the GLOW2 study. Eur Respir J. 2012;40:1106–14.
35. Mahler DA, D'Urzo A, Bateman ED, et al. Concurrent use of indacaterol plus tiotropium in patients with COPD provides superior bronchodilation compared with tiotropium alone: a randomised, double-blind comparison. Thorax. 2012;67:781–8.
36. Novartis. Efficacy and Safety of Indacaterol Plus Tiotropium Versus Tiotropium Alone in Patients With Chronic Obstructive Pulmonary Disease (INTRUST1) (NCT00846586). 2011. [Available from: http://www.clinicaltrials.gov/ct2/show/results/NCT00846586]. Accessed 2 Oct 2015.
37. Novartis. A randomized, double-blind, controlled, parallel group, 12-week treatment study to compare the efficacy and safety of the combination of indacaterol 150 µg once daily with open label tiotropium 18 µg once daily versus open label tiotropium 18 µg once daily in patients with moderate to severe chronic obstructive pulmonary disease. 2009. [Available from: https://www.clinicaltrialsregister.eu/ctr-search/search?query=eudract_number:2008-006976-31]. Accessed 8 Oct 2015.
38. Novartis. Efficacy and Safety of Indacaterol Plus Tiotropium Versus Tiotropium Alone in Patients With Chronic Obstructive Pulmonary Disease (INTRUST2) (NCT00877383). 2011. [Available from: http://clinicaltrials.gov/ct2/show/NCT00877383]. Accessed 2 Oct 2015.
39. Novartis. A randomized, double-blind, controlled, parallel group, 12-week treatment study to compare the efficacy and safety of the combination of indacaterol 150 µg once daily with open label tiotropium 18 µg once daily versus open label tiotropium 18 µg once daily in patients with moderate-to-severe chronic obstructive pulmonary disease. 2009. [Available from: https://www.clinicaltrialsregister.eu/ctr-search/search?query=2008-008447-26]. Accessed 8 Oct 2015.
40. Moita J, Barbara C, Cardoso J, et al. Tiotropium improves FEV1 in patients with COPD irrespective of smoking status. Pulm Pharmacol Ther. 2008;21:146–51.
41. Niewoehner DE, Rice K, Cote C, et al. Prevention of exacerbations of chronic obstructive pulmonary disease with tiotropium, a once-daily inhaled anticholinergic bronchodilator: a randomized trial. Ann Intern Med. 2005;143:317–26.
42. Singh D, Ferguson GT, Bolitschek J, et al. Tiotropium + olodaterol shows clinically meaningful improvements in quality of life. Respir Med. 2015;109:1312–9.
43. Buhl R, Gessner C, Schuermann W, et al. Efficacy and safety of once-daily QVA149 compared with the free combination of once-daily tiotropium plus twice-daily formoterol in patients with moderate-to-severe COPD (QUANTIFY): a randomised, non-inferiority study. Thorax. 2015;70:311–9.
44. Bateman ED, Ferguson GT, Barnes N, et al. Dual bronchodilation with QVA149 versus single bronchodilator therapy: the SHINE study. Eur Respir J. 2013;42:1484–94.
45. Novartis. A Study to Assess the Efficacy, Safety and Tolerability of Once-daily (q.d.) QVA149 in Patients With Moderate to Severe Chronic Obstructive Pulmonary Disease (COPD) (NCT01202188). 2013. [Available from: http://ClinicalTrials.gov/show/NCT01202188]. Accessed 2 Oct 2015.

46. Novartis. A 26-week treatment multi-center, randomized, doubleblind, parallel-group, placebo and active controlled (open label) study to assess the efficacy, safety and tolerability of QVA149 (110/50 µg q.d.) in patients with moderate to severe chronic obstructive pulmonary disease (COPD). 2013. [Available from: https://www.clinicaltrialsregister.eu/ctr-search/search?query=2009-017772-25]. Accessed 8 Oct 2015.
47. Wedzicha JA, Decramer M, Ficker JH, et al. Analysis of chronic obstructive pulmonary disease exacerbations with the dual bronchodilator QVA149 compared with glycopyrronium and tiotropium (SPARK): a randomised, double-blind, parallel-group study. Lancet Respir Med. 2013;1:199–209.
48. Novartis. Effect of QVA149 Versus NVA237 and Tiotropium on Chronic Obstructive Pulmonary Disorder (COPD) Exacerbations (NCT01120691). 2013. [Available from: http://ClinicalTrials.gov/show/NCT01120691]. Accessed 2 Oct 2015.
49. Novartis. A 64-week treatment, multi-center, randomized, double-blind, parallel-group, active controlled study to evaluate the effect of QVA149 (110/50 µg o.d.) vs NVA237 (50 µg o.d.) and open-label tiotropium (18 µg o.d.) on COPD exacerbations in patients with severe to very severe chronic obstructive pulmonary disease (COPD). 2014. [Available from: https://www.clinicaltrialsregister.eu/ctr-search/search?query=2009-013256-69]. Accessed 8 Oct 2015.
50. Tashkin DP, Pearle J, Iezzoni D, Varghese ST. Formoterol and tiotropium compared with tiotropium alone for treatment of COPD. COPD. 2009;6:17–25.
51. Wise RA, Anzueto A, Cotton D, et al. Tiotropium Respimat inhaler and the risk of death in COPD. N Engl J Med. 2013;369:1491–501.
52. Anzueto A, Wise R, Calverley P, et al. The Tiotropium Safety and Performance in Respimat® (TIOSPIR®) Trial: Spirometry Outcomes. Respir Res. 2015;16:107.
53. Tonnel AB, Perez T, Grosbois JM, et al. Effect of tiotropium on health-related quality of life as a primary efficacy endpoint in COPD. Int J Chron Obstruct Pulmon Dis. 2008;3:301–10.
54. Ferguson GT, Flezar M, Korn S, et al. Efficacy of Tiotropium + Olodaterol in Patients with Chronic Obstructive Pulmonary Disease by Initial Disease Severity and Treatment Intensity: A Post Hoc Analysis. Adv Ther. 2015;32:523–36.
55. Buhl R, Maltais F, Abrahams R, et al. Tiotropium and olodaterol fixed-dose combination versus mono-components in COPD (GOLD 2-4). Eur Respir J. 2015;45:969–79.
56. Troosters T, Sciurba FC, Decramer M, et al. Tiotropium in patients with moderate COPD naive to maintenance therapy: a randomised placebo-controlled trial. NPJ Prim Care Respir Med. 2014;24:14003.
57. Tashkin DP, Celli B, Senn S, et al. A 4-year trial of tiotropium in chronic obstructive pulmonary disease. N Engl J Med. 2008;359:1543–54.
58. Tashkin DP, Li N, Kleerup EC, et al. Acute bronchodilator responses decline progressively over 4 years in patients with moderate to very severe COPD. Respir Res. 2014;15:102.
59. Buhl R, Welte T, Vogelmeier C, et al. [Early treatment of COPD with tiotropium]. Pneumologie. 2012;66:589–95.
60. Tashkin DP, Celli BR, Decramer M, Lystig T, Liu D, Kesten S. Efficacy of tiotropium in COPD patients with FEV1 ≥ 60% participating in the UPLIFT® trial. COPD. 2012;9:289–96.
61. Celli B, Decramer M, Kesten S, et al. Mortality in the 4-year trial of tiotropium (UPLIFT) in patients with chronic obstructive pulmonary disease. Am J Respir Crit Care Med. 2009;180:948–55.
62. Verkindre C, Bart F, Aguilaniu B, et al. The effect of tiotropium on hyperinflation and exercise capacity in chronic obstructive pulmonary disease. Respiration. 2006;73:420–7.
63. Vogelmeier C, Kardos P, Harari S, Gans SJ, Stenglein S, Thirlwell J. Formoterol mono- and combination therapy with tiotropium in patients with COPD: a 6-month study. Respir Med. 2008;102:1511–20.
64. Voshaar T, Lapidus R, Maleki-Yazdi R, et al. A randomized study of tiotropium Respimat Soft Mist inhaler vs. ipratropium pMDI in COPD. Respir Med. 2008;102:32–41.
